# ATM and PRDM9 regulate SPO11-bound recombination intermediates during meiosis

**DOI:** 10.1038/s41467-020-14654-w

**Published:** 2020-02-12

**Authors:** Jacob Paiano, Wei Wu, Shintaro Yamada, Nicholas Sciascia, Elsa Callen, Ana Paola Cotrim, Rajashree A. Deshpande, Yaakov Maman, Amanda Day, Tanya T. Paull, André Nussenzweig

**Affiliations:** 1 Laboratory of Genome Integrity, National Cancer Institute, NIH, Bethesda, MD USA; 2 Immunology Graduate Group, University of Pennsylvania, Philadelphia, PA USA; 3 Molecular Biology Program, Memorial Sloan Kettering Cancer Center, New York, NY USA; 4 Department of Radiation Genetics, Graduate School of Medicine, Kyoto University, Kyoto, 606-8501 Japan; 5 Institute for Biomedical Sciences, George Washington University, Washington, DC USA; 6 The Howard Hughes Medical Institute and The University of Texas at Austin, Austin, TX 78712 USA; 7 The Department of Molecular Biosciences, The University of Texas at Austin, Austin, TX 78712 USA

**Keywords:** DNA damage and repair, DNA recombination

## Abstract

Meiotic recombination is initiated by SPO11-induced double-strand breaks (DSBs). In most mammals, the methyltransferase PRDM9 guides SPO11 targeting, and the ATM kinase controls meiotic DSB numbers. Following MRE11 nuclease removal of SPO11, the DSB is resected and loaded with DMC1 filaments for homolog invasion. Here, we demonstrate the direct detection of meiotic DSBs and resection using END-seq on mouse spermatocytes with low sample input. We find that DMC1 limits both minimum and maximum resection lengths, whereas 53BP1, BRCA1 and EXO1 play surprisingly minimal roles. Through enzymatic modifications to END-seq, we identify a SPO11-bound meiotic recombination intermediate (SPO11-RI) present at all hotspots. We propose that SPO11-RI forms because chromatin-bound PRDM9 asymmetrically blocks MRE11 from releasing SPO11. In *Atm*^*–/–*^ spermatocytes, trapped SPO11 cleavage complexes accumulate due to defective MRE11 initiation of resection. Thus, in addition to governing SPO11 breakage, ATM and PRDM9 are critical local regulators of mammalian SPO11 processing.

## Abstract (web summary)

Recombination requires DNA break formation by SPO11, following which SPO11 is thought to be released. Here, the authors show that meiotic hotspots retain SPO11 through a recombination intermediate dependent on the methyltransferase PRDM9, and that the ATM kinase governs the release of SPO11.

## Introduction

**Fig. 1:**
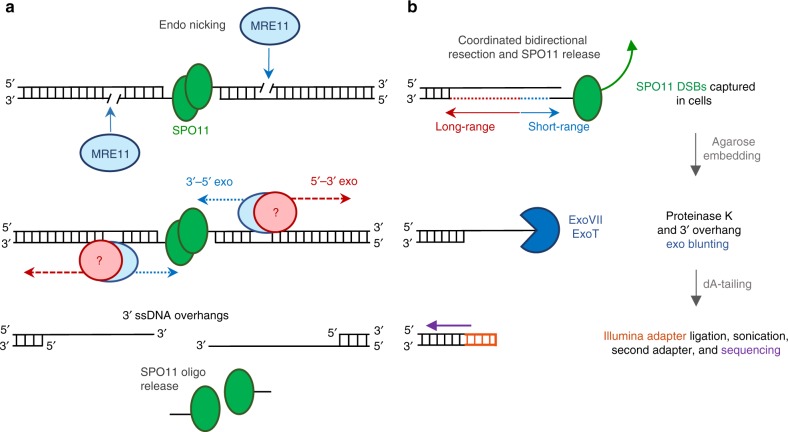
SPO11 generates meiotic DSBs that are detectable by END-seq. **a** Illustration of meiotic break generation and processing. SPO11 induces a double-strand break (DSB) and remains covalently bound to both DNA ends. MRE11 recognizes the DSB and induces a nick on the SPO11-bound strand. Tightly coordinated short-range 3′−5′ resection by MRE11 and long-range 5′−3′ resection by an unknown nuclease generates 3′ overhangs for homology search. MRE11 activity releases SPO11 bound to short oligonucleotides (SPO11 oligos). **b** Brief schematic of END-seq detection of SPO11 DSBs (only one side of the DSB is shown for simplicity). In vivo processing of SPO11 by coordinated bidirectional resection removes covalently bound SPO11 and produces a 3′ overhang present at the time of END-seq preparation and agarose cell embedding. Initial END-seq processing degrades all proteins by proteinase K and blunts ssDNA overhangs by in vitro nuclease digestion (dark blue). Once fully blunted and dA-tailed, the DNA end is ligated to a biotinylated Illumina sequencing adapter (orange), sheared, and streptavidin captured. A second Illumina adapter is ligated at the other end of the sonicated fragment after end repair and sequenced.

Recombination between homologous chromosomes during meiosis requires DNA double-strand break (DSB) formation by the topoisomerase-like protein SPO11^1^. After cutting, SPO11 remains covalently bound to a two-nucleotide, 5′ overhang at both ends of the DNA via phosphotyrosyl linkage. Recombination then begins with the processing of SPO11-bound DSBs into resected 3′ single-stranded DNA (ssDNA) tails that preferentially invade the homologous chromosome by the recombinases DMC1 and RAD51. Studies in budding yeast *Saccharomyces cerevisiae* determined that the MRE11/RAD50/NBS1 (MRN) complex detects SPO11 and cooperates with Sae2 to produce a nick on the SPO11-bound strand via MRE11 endonuclease activity^2^. The nick serves as an entry point for both short-range MRE11 3′−5′ exonuclease activity to degrade back to the DSB, thereby removing covalently bound SPO11 attached to a ssDNA oligonucleotide, as well as for more-extensive long-range processing of 5′ strands (Fig. 1a)^2^. In budding yeast, Exo1 nuclease is uniquely responsible for this long-range 5′−3′ resection^3^. Moreover, short- and long-range resection are tightly coupled in a single processive reaction (Fig. 1a). As a result, meiotic DSBs are maximally resected as soon as they appear and unresected SPO11-bound DSBs are extremely rare^4–6^. Although ATM has been shown to regulate DSB numbers and locations^7,8^, it remains unclear whether it also functions downstream in regulating SPO11 processing and resection.

Distinct from yeast, DSB hotspots in mice and humans are determined by the DNA-binding specificity of the PRDM9 methyltransferase^9^. Besides positioning DSBs, PRDM9-binding activity also reorganizes nucleosomes in a manner that creates a nucleosome-depleted region (NDR) within which DSBs and PRDM9 itself are centered^10^. Moreover, PRDM9 has been suggested to have a role in DSB repair post cleavage^11,12^. Crossover resolution is facilitated by PRDM9 binding symmetrically to the template (uncut) homolog, which generates a NDR within which the DSB-initiating chromosome can stably engage^13–15^. If PRDM9 remains bound to DNA post-cleavage, it is possible that it could influence the downstream processing events that facilitate synapsis and crossover recombination.

Here, we demonstrate that ATM regulates multiple steps of initial SPO11 processing, including the activation of MRE11, the coordination of short- and long-range resection, and the assurance of minimal SPO11 cutting per hotspot. However, we find that ATM-mediated SPO11 processing can be hindered by the binding of PRDM9 to hotspots post-SPO11 cutting. We suggest that PRDM9 acts as a physical barrier to MRE11 activity, preventing SPO11 release during the early stages of homolog engagement. This generates a PRDM9-dependent SPO11-bound recombination intermediate (SPO11-RI). We propose that SPO11-RI may favor high-fidelity homologous recombination by facilitating crossover (CO) and non-crossover (NCO) events.

## Results

### END-seq robustly detects mouse meiotic DSB hotspots

To probe the early steps of mouse meitotic recombination, we utilized END-seq^16–18^. In this method, a sequencing adapter is ligated to each end of a DNA break inside an agarose plug after a combination of nucleases ExoVII and ExoT removes ssDNA overhangs. As a result, sequencing reads begin at the terminal end of physiological resection, resulting in libraries of ssDNA-DSB junctions (Fig. 1b). These exonucleases can be used to detect DSB termini that are either protein-bound or protein-free. For example, etoposide-induced DSBs, which are covalently attached to topoisomerase 2 (TOP2) via an active site tyrosine at the 5′-termini, require ExoVII to remove covalently bound TOP2^16^, whereas ExoT can only blunt protein-free overhangs, resulting in a ligatable DNA end^18^. Like TOP2, the topoisomerase-like protein SPO11 remains attached to DSB 5′-ends prior to release by MRE11-mediated nicking as short 20–40 bp oligonucleotides (SPO11 oligos)^7,19^ (Fig. 1a).

**Fig. 2:**
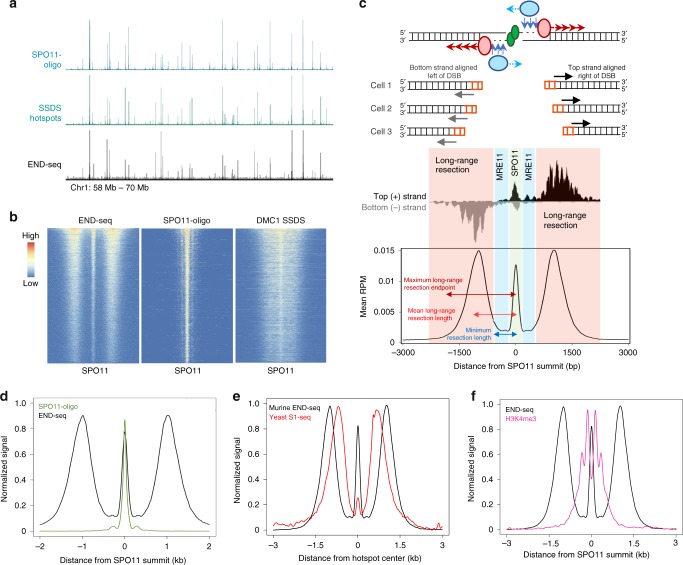
END-seq correlates with previous hotspot mapping techniques and uncovers a uniform pattern of SPO11 processing at all hotspots. **a** Representative genome browser profiles of meiotic hotspots for SPO11-oligo sequencing, DMC1 SSDS, and END-seq. Browser axis scales are adjusted between techniques to show both hot and cold hotspots simultaneously. **b** Heatmaps of END-seq, SPO11-oligo, and SSDS ± 2.5 kb around hotspot centers (determined by SPO11-oligo summits), ordered by total read count of END-seq, for top 5000 END-seq breaks. WT END-seq peaks are provided in Supplementary Data 1. **c** Schematic of END-seq break pattern, consisting of (1) a central peak at the SPO11 break site (2) a read-less gap produced by MRE11-mediated short-range resection and minimum distance of long-range resection, and (3) distal reads at the terminal ends of long-range resection. This pattern is evident at individual hotspots (middle, chr1:68488000–68491500) and when signal from all hotspots is aggregated (bottom). Minimum resection lengths are calculated by the absence of sequencing reads (blue highlighted region); mean and maximum long-range resection are calculated by the average and most distal reads from the DSB, respectively (red highlighted region). **d** END-seq central peak and SPO11-oligo reads are coincident as evidenced by aggregated signal around SPO11-oligo summits (normalized to the same height). Both the primary SPO11 peak and adjacent secondary peaks are apparent. **e** Aggregate signal comparison of murine meiotic END-seq around SPO11-oligo summits and yeast meiotic S1-seq around yeast hotspot centers. **f** Aggregated END-seq and H3K4me3 ChIP-seq signal (normalized to the same height).

We assayed spermatocytes from juvenile mouse testes during the first wave of semi-synchronous meiosis I. We embedded spermatocytes from 20 pooled, 12–14 dpp C57BL/6 J (B6) mice in agarose plugs and blunted meiotic ssDNA overhangs with ExoVII and ExoT before ligating sequencing adapters (Fig. 1b). Approximately 5000 reproducible broken hotspots were called with a threshold of at least 2.5-fold enrichment (Supplementary Fig. 1a, Supplementary Data 1). We overlapped these END-seq peaks with previously reported B6 meiotic hotspots determined by SPO11-oligo sequencing and DMC1 single-strand DNA sequencing (SSDS)^8,11,20^. By visual inspection and correlative analysis, both END-seq break location and peak intensity overlapped with SPO11 oligos and SSDS, which accounted for 97% and 98% of END-seq peaks, respectively (Fig. 2a, Supplementary Fig. 1b, c). END-seq therefore provides a map of directly detected meiotic DSB resection in mammals.

Using the 2.5-fold-enrichment criteria, END-seq peaks called a third of the total hotspots determined by SPO11-oligo seq or SSDS (Supplementary Fig. 1b, c). However, END-seq peaks that did not meet this cutoff were nevertheless associated with previously mapped hotspots. For example, 66% of SPO11-oligo loci that were “END-seq negative” showed the same DSB detection pattern when these END-seq reads were aggregated (Supplementary Fig. 1d, e). Moreover, these END-seq-negative SPO11-oligo sites show a significant reduction in SPO11-oligo reads (Supplementary Fig. 1f), indicating that these are the coldest meiotic hotspots. We conclude that END-seq detects breakage at all ~ 15,000 previously mapped hotspots, yet the more frequently broken top 33% of hotspots yield the most robust signal. Therefore, unless otherwise stated, the subsequent analyses were performed on the 5000 strongest END-seq breaks.

### The landscape of mouse meiotic DSB resection

END-seq captured a strikingly uniform pattern of breakage at all sites that consisted of a strong central peak directly at the site of SPO11 cutting with an accumulation of reads flanking the cut site at a defined distance away (Fig. 2b, c). We interpret the central peak to be the direct detection of SPO11 breakage while adjacent, distal reads reflect minimum and maximum resection endpoints in the population of spermatocytes. Reads comprising the central peak are entirely coincident with SPO11-oligo mapping and reflect a subset of breaks within the spermatocyte population that has not yet released covalently bound SPO11 from the DSB (Fig. 2d). As discussed later, the central peak in WT cells is dependent on engagement of the cut chromosome with the uncut homolog, and therefore represents unreleased SPO11 associated with recombination.

END-seq detects the terminal end of physiologic resection after in vitro blunting of the 3′ overhang, with the first nucleotide sequenced corresponding to the position of the ssDNA-DSB junction (Figs. 2c, 1b)^18^. If bidirectional resection of DNA-bound SPO11 by short- and long-range resection machineries are entirely coordinated as in yeast^4–6^, then the ssDNA-DSB junction will always be beyond the most distal MRE11 nick and correspond to the terminal end of long-range resection (Figs. 2c, 1b). In this case, the location of short-range 3′−5′ MRE11 exonuclease activity would not yield any sequencing reads as it does not operate independently of 5′−3′ resection (Figs. 2c, 1b). Indeed, at every hotspot, we observed a read-less “gap” in resection, consistent with the tight coupling of resection initiation (by MRE11) and 5′−3′ extension by the long-range resection machinery (Fig. 2b, c). The length of this gap reflects the minimum resection endpoints in the spermatocyte population and corresponds to the maximum distance from the DSB at which MRE11 nicks the strand plus any constant, minimum distance that 5′−3′ exonucleases traverse. This pattern is reminiscent of S1 nuclease detection of meiotic recombination in yeast^6^ (Fig. 2e). Thus, both SPO11 cutting and its initial processing by coordinated resection mechanisms are highly conserved evolutionary features of meiotic recombination that span unicellular eukaryotes to mammals.

The gap size was extremely uniform at all hotspots, with mean maximum distance of 647 nts (Supplementary Fig. 2a), and was largely restricted to the two-nucleosome H3K4/K36me3 signal surrounding SPO11 cut sites as determined by ChIP-seq (Fig. 2f, Supplementary Fig. 2b). Thus, minimum resection distances correlate well with PRDM9-mediated methylated histone deposition^10^. Interestingly, the majority of CO and NCO boundaries in mice are also restricted to this region, rarely extending beyond the 650 bp gap (Supplementary Fig. 2c)^15,21^. Because minimum resection correlated well with methylated histones, we asked if long-range resection endpoints were similarly well positioned. Between two replicate END-seq samples, we found high correlation in the pattern of resection endpoints among the hotter hotspots detected with recurrent subpeaks apparent at a mean distance of 210 nucleotides (Supplementary Fig. 2d, e). This suggests that nucleosome occupancy far from the break site may influence long-range resection endpoints.

### Meiotic DSB hotspots are detectable in a single mouse

**Fig. 3:**
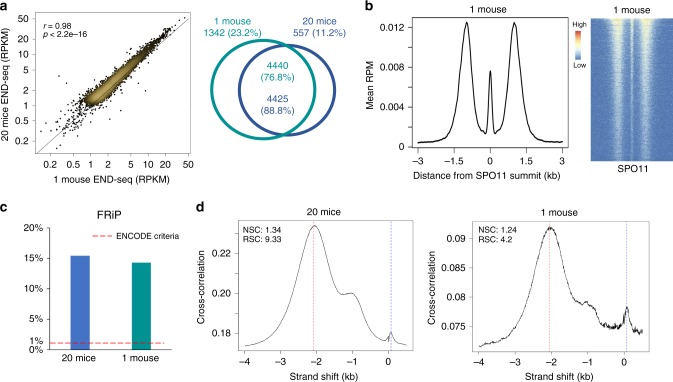
END-seq detects meiotic DSBs in a single mouse. **a** Left panel: correlation (Pearson’s *r*) between END-seq RPKM from 20 mice versus RPKM from one 12 dpp mouse ± 3 kb around SPO11 summits. Right panel: Venn diagram overlap of called peaks between 20 mice and one mouse. *P* value < 2.2e-16, Fisher’s exact test. **b** Left panel: Full END-seq break pattern in aggregated signal from one mouse, centered around SPO11 oligos. Right panel: heatmap of END-seq signal from one mouse in a ± 3 kb window around SPO11 oligos, ordered by total read count. **c** FRiP values for 20 mice versus one mouse. Recommended ENCODE value denoted by dotted red line. **d** Cross-correlation plot profiles for 20 mice and one mouse. The plot shows Pearson cross-correlations (CCs, *y* axis) of read intensities between the plus strand and the minus strand, after shifting minus strand (*x* axis). One peak corresponds to read length (CCread, blue dash line) and the other one corresponds to the fragment length (CCfrag, red dash line). Normalized strand coefficient (NSC) is CCfrag divided by minimal CC value (CCmin) and relative strand coefficient (RSC) is the ratio of CCfrag-CCmin divided by CCread-CCmin. Higher NSC and RSC values mean more enrichment. ENCODE’s recommendation for ChIP-seq: NSC ≥ 1.05 and RSC ≥ 0.8.

To determine the sensitivity of the method, we compared 20 pooled juvenile mice to a library made from a single 12 dpp mouse. Both samples called ~ 5000 peaks, with 77–89% shared breaks with highly correlated (*r* = 0.98) END-seq intensities (Fig. 3a). Importantly, the break pattern at individual hotspots was fully retained in testes from a single mouse (Fig. 3b). To compare signal-to-noise ratio (S/N) in the two libraries, we followed ENCODE’s assessment of fraction of reads in peaks (FRiP) and cross-correlation profiles (CCPs) for ChIP-seq data sets^22,23^. FRiP and CCP values for both libraries exceeded ENCODE’s criteria for signal-to-noise ratios (Fig. 3c, d). We conclude that END-seq can accurately assess individual meiotic DSB locations and processing with remarkably little biological material. This high sensitivity bypasses the limitations of other hotspot mapping methods that require either impractical quantities of mice (SPO11-oligo seq) or the availability of species-specific, high-quality antibodies (DMC1 SSDS).

### Increased breakage in *Atm*^–/–^ spermatocytes

ATM is thought to negatively regulate SPO11 cutting, and in its absence, hotspot breakage and SPO11 oligos have been shown to markedly increase in mice^7^. This ultimately results in early meiotic arrest and apoptosis of *Atm*^–/–^ spermatocytes that carry an excess of unrepaired DSBs^7^. To further validate this model using direct, genome-wide detection, we performed END-seq on *Atm*^–/–^ spermatocytes that were backcrossed 11 times to the B6 background. Indeed, *Atm*^–/–^ END-seq detected 99% of WT END-seq breaks while calling ~ 6300 more peaks (Supplementary Fig. 3a, Supplementary Data 2). All *Atm*^–/–^ breaks overlapped better with SPO11 oligos and SSDS sites and showed amplified signal over WT at weak SSDS hotspots (Supplementary Fig. 3b–d). These data confirm previous reports that colder hotspots become preferentially hotter in the absence of ATM owing to a loss of negative feedback of SPO11 cutting (Supplementary Fig. 3e)^7,8^. To quantify this increase, we added a spike-in normalization control to END-seq plugs, consisting of a G1-arrested Ableson-transformed murine pre-B cell line (*Lig4*^*–/–*^) carrying a single zinc-finger-induced DSB at the TCRβ enhancer^16^, which was mixed in at a 2% frequency with bulk testiscular cells. After normalizing END-seq reads at all ~ 15,000 SPO11-oligo hotspots using the signal at the TCRβ enhancer, we found that *Atm*^–/–^ cells harbor a 4.5-fold increase in total breaks over WT. As discussed later, increased SPO11 double-cutting within the same hotspot may account for the 10-fold increase in SPO11 oligos released in *Atm*^–/–^ spermatocytes;^7^ yet END-seq would detect double-cutting as a single event, yielding a 4.5-fold increase in DSBs.

Unexpectedly we found that 7–16% (700–1800) of break sites were not shared in WT SSDS or SPO11-oligo maps, respectively (Supplementary Fig. 3b, c). Among these were several hundred promoter breaks, typically associated as “default” hotspots in organisms lacking PRDM9 (Supplementary Fig. 3f). Indeed, these break locations are among the hottest *Prdm9*^–/–^ sites as determined by SSDS (Supplementary Fig. 3g)^11,24^. Thus, in the absence of ATM, colder hotspots and default hotspots become increasingly broken. This is consistent with previous observations that ATM-null spermatocytes have increased SPO11-oligo mapping at PRDM9-independent hotspots^14^.

### Minimal roles for EXO1, BRCA1, and 53BP1 during resection

**Fig. 4:**
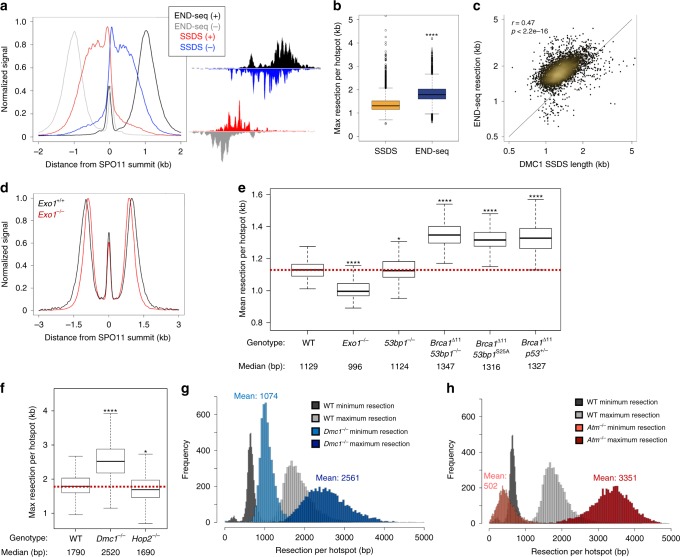
END-seq accurately measures hotspot resection lengths and elucidates resection regulation. **a** Top (+) and bottom (–) strand distributions of END-seq and SSDS show increased resection detection by END-seq (left, signal normalized to the same height) that is evident at individual hotspots (right). **b** Boxplot of END-seq vs SSDS maximum resection per hotspot. ****p < 1e-10; *t* test. **c** Correlation (Pearson’s *r*) of maximum resection endpoints detected by END-seq and SSDS. **d** Aggregate plot of END-seq signal in WT and *Exo1*^–/–^ spermatocytes (signal normalized to the same height) around SPO11 oligos. **e** Boxplots of mean resection (as defined in Fig. 2c) between WT, *Exo1*^–/–^, *53bp1*^–/–^, *Brca1*^*Δ11*^*53bp1*^–/–^, *Brca1*^*Δ11*^*53bp1*^*S25A*^ and *Brca1*^*Δ11*^*p53*^*+/–*^ at top 250 hotspots. *p < 0.01; ****p < 1e-10; *t* test with mu = 10 (mu is estimated as standard deviation of WT replicates). **f** Boxplots of maximum resection endpoints between WT, *Dmc1*^–/–^, and *Hop2*^–/–^ at top 5000 hotspots. *p < 0.01; ****p < 1e-10; *t* test with mu = 43 (mu is estimated as standard deviation of WT replicates). **g**, **h** Histogram distributions comparing either WT and *Dmc1*^–/–^ **g** or WT and *Atm*^–/–^ **h** END-seq minimum and maximum resection endpoints at top 5000 hotspots. Mean values (bp) are listed.

Long-range resection endpoints showed greater variation (1–3 kb) relative to the minimum resection gap region (0.4–1 kb) (Supplementary Fig. 2a). Previous studies estimated total mammalian meiotic resection lengths based on the extent of DMC1 bound to ssDNA overhangs as measured by DMC1 SSDS^8^. Strikingly, we found that maximum resection endpoints extended significantly farther at all hotspots than DMC1-bound ssDNA (Fig. 4a, b). ssDNA occupied by DMC1 ranged from 800 to 2700 nts, whereas maximum long-range resection lengths (defined in Fig. 2c) determined by END-seq were 1.2–1.6-fold greater (Fig. 4b, c). These data indicate that DMC1 binds to only a portion of the available ssDNA and underestimates the total extent of meiotic resection. One potential explanation is that single tracks of ssDNA can be co-occupied by DMC1 and RAD51 filaments with DMC1 loaded more proximally to the break site than RAD51^25^. Although unlikely, this difference may also be due to technical limitations in SSDS library preparation that relies on microhomology-mediated hairpin formations naturally present in ssDNA tracks^26^.

Because the resection pattern in mouse mirrored so closely that observed in yeast (Fig. 2e)^6^, we wanted to know whether they utilized the same long-range end-processing machineries. Exo1-deficient yeast exhibit total loss of 5′−3′ long-range resection^3^. In contrast, EXO1 and DNA2 act redundantly in yeast vegetative and mammalian somatic cells to mediate end resection^27–29^. Surprisingly, END-seq analysis of juvenile *Exo1*^–/–^ spermatocytes revealed that long-range resection was largely intact (Fig. 4d). When averaged genome-wide, we found a small, though significant, reduction in long-range resection distance in *Exo1*^–/–^ cells (median resection tract: WT: 1,129 nts vs. *Exo1*^–/–^: 996 nts (Fig. 4e). We therefore conclude that compared with yeast, mammalian meiosis has evolved additional mechanisms to achieve extensive 3′ overhangs, perhaps through utilization of redundant DNA2 exonuclease activity^27–29^.

In somatic interphase cells, 53BP1 has been shown to inhibit long-range resection of DSBs^30^. One principal role of BRCA1 is to counteract 53BP1’s block to resection in S phase, possibly by excluding it from chromatin proximal to DNA damage sites^31^. In addition, BRCA1 acts post-resection to load the RAD51 recombinase onto 3′ ssDNA^32^. Despite these extensive studies demonstrating their importance in somatic cells, the roles of 53BP1 and BRCA1 in regulating meiotic resection are unknown. We therefore performed END-seq on *53bp1*^–/–^ and BRCA1-deficient spermatocytes and measured overall resection lengths. In striking contrast to 53BP1-deficient somatic cells in which DSB resection is consistently and acutely increased^18,30,33^, we found that resection lengths were comparable at all meiotic hotspots in *53bp1*^–/–^ and WT spermatocytes (Fig. 4e).

We then assayed *Brca1*^*Δ11*^*p53*^+/–^ mice, which exhibit known defects in BRCA1 function, yet are alive owing to partial p53 apoptotic suppression^34^. Contrary to our expectations^30,33^, *Brca1*^*Δ11*^*p53*^+/–^ spermatocytes showed a mild increase in DSB resection relative to WT controls (Fig. 4e). Moreover, *Brca1*^*Δ11*^*53bp1*^*–/–*^ and *Brca1*^*Δ11*^*53bp1*^*S25A*^ spermatocytes^33^ exhibited a similar increase in resection as *Brca1*^*Δ11*^*p53*^+/−^ spermatocytes (Fig. 4e). Based on these findings, we conclude that in contrast to their well-defined antagonistic relationship during DSB processing in interphase cells, 53BP1 does not inhibit resection while BRCA1 does not promote resection during meiotic recombination. As discussed below, one reason why end resection increases (rather than decreases) in BRCA1-deficient cells may be owing to inefficient loading of the recombinase. Consistent with this, *Brca1*^*Δ11*^*p53*^+/–^ spermatocytes have reduced RAD51 and DMC1 focus counts^35^.

### RAD51, DMC1, and ATM negatively regulate long-range resection

In budding yeast, cells lacking DMC1 show hyper-resection, perhaps owing to a negative feedback on resection mediated by recombinase loading^4,6^. Given our results that BRCA1-deficient spermatocytes exhibited increased resection (Fig. 4e) associated with defective RAD51/DMC1 foci^35^, we hypothesized that RAD51 or DMC1 loading onto ssDNA might limit long-range resection. Consistent with this, *Dmc1*^–/–^ spermatocytes showed substantially increased resection relative to WT at all hotspots (Fig. 4f, g and Supplementary Fig. 4a). Minimum resection lengths also increased in *Dmc1*^–/–^ cells by ~ 400 nts relative to WT (Fig. 4g and Supplementary Fig. 4a).

HOP2 protein is required for proper homolog pairing after loading of DMC1/RAD51 recombinases^36^. In *Hop2*^−/−^ spermatocytes, resection lengths were similar to WT (Fig. 4f). Thus, recombinase loading post-resection, independent of strand invasion, limits both the minimum and maximum length that the long-range resection machinery processes DSBs. DMC1/RAD51 loading onto ssDNA might limit resection by preventing the re-initiation of exonucleases on already resected ssDNA.

ATM negatively regulates DSB induction by SPO11^7^. Despite the fact that *Atm*^–/–^spermatocytes harbor a 10-fold increase in SPO11 oligos^7^, RAD51/DMC1 foci form at similar levels as in WT, whereas RPA foci counts are increased in mutant cells^37^. One possible reason why the number of foci does not correlate with the large increase in DSBs could be that recombinase level or activity is limiting filament formation. In this scenario, when excess DSBs are generated in an ATM-null background, the pool of RAD51 and/or DMC1 is insufficient to load onto all ssDNA regions. In accord with this hypothesis, the mean maximum resection lengths increased almost twofold in *Atm*^–/–^ spermatocytes relative to WT (WT: 1845 nts. vs. ATM: 3351 nts; Fig. 4h). Moreover, in ATM knockouts, resection lengths were significantly greater and more widely distributed than even DMC1 knockouts (Fig. 4g, h), perhaps owing to combined deficiency in RAD51 and DMC1 loading onto ssDNA^37^. In accord with the idea that increased DSB numbers rather than deficiency in ATM signaling contributes to the hyper-resection phenotype in the ATM-null background, S1-seq analysis of *Atm*^–/–^*Spo11*^+/–^ spermatocytes, in which SPO11-oligo complexes are reduced by half^7^, revealed normal resection lengths^38^. Together these data support the idea that the availability of recombinases to form filaments is limiting and insufficient when there is excessive SPO11 cutting.

### ATM coordinates short- and long-range resection

**Fig. 5:**
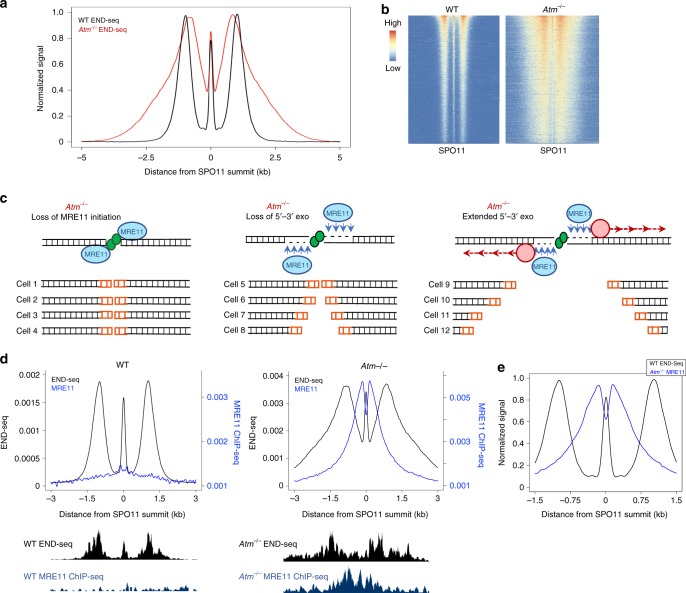
ATM coordinates multiple levels of resection machinery. **a** Aggregate plots of END-seq signal in WT vs *Atm*^–/–^ at top 5000 WT breaks around SPO11-oligo summits. Signals are normalized to the same height for DSB pattern clarity. **b** Heatmaps of WT and *Atm*^–/–^ END-seq ± 5 kb around SPO11-oligo summits for top 5000 WT hotspots. Signals are normalized to spike-in control to show increased *Atm*^–/–^ break intensity per hotspot. **c** Illustrations of ATM’s multiple roles in coordinating resection that gives overall heterogeneous END-seq pattern in **a**. Left: MRE11 is recruited yet not activated in a subset of cells, resulting in reads directly at SPO11 DSB within the population. Middle: MRE11 is activated in another subset of cells, yet long-range resection is not sufficiently initiated, resulting in reads from only MRE11 nicking, and perhaps 3′−5′ exonuclease activity, flanking the DSB in regions that are read-less in WT. Right: resection is properly initiated in another subset of cells, yet long-range resection travels significantly farther than in WT. **d** Aggregated END-seq signal and MRE11 ChIP-seq RPM in WT (left) and *Atm*^*–/–*^ (right). To fairly compare ChIP-seq signal between WT and *Atm*^*–/–*^, MRE11 scale is proportional to spike-in normalized END-seq RPM for each genotype. Individual hotspot examples (chr12:34,592,264-34,598,265) are shown below. Note that decreased MRE11 coverage is observed within NDR of *Atm*^*–/–*^. **e** Aggregate plot overlapping WT END-seq and *Atm*^–/–^ MRE11 ChIP-seq, normalized to the same height. MRE11 shows prominent localization to the WT END-seq read-less gap.

In addition to the hyper-resection observed at a subset of breaks, distinct boundaries between the short- and long-range resection practically disappeared in *Atm*^–/–^ spermatocytes. This resulted in reads mapping within the gap region (Fig. 5a, b), in contrast to WT that always generates ssDNA-DSB junctions beyond the most distal MRE11 nick. The decreased minimum resection could indicate that ATM regulates the tightly coupled activities of short-range and long-range resection (Fig. 5c).

If some DSBs remain unresected in ATM-deficient spermatocytes, we would predict that MRE11 would accumulate at these unprocessed DNA ends. Consistent with this hypothesis, ChIP-seq for MRE11 revealed significant chromatin binding at all hotspots in *Atm*^*–/–*^ cells (Fig. 5d, right, Supplementary Fig. 4b), which correlated with END-seq intensity (Supplementary Fig. 4c). This likely reflects a role for ATM in promoting normal MRE11 resection initiation as suggested for Tel1 in yeast^6^. In contrast, we detected no signal in similarly broken regions in WT cells (Fig. 5d, left), indicative of completed resection. Strikingly, the MRE11 signal in ATM-null cells was contained within the WT read-less gap (Fig. 5d, e, Supplementary Fig. 4b). This lends further support to the idea that the gap reflects MRE11-dependent end-processing. Interestingly, MRE11 binding greatly diminished in the small central H3K4me3/K36me3 nucleosome-depleted area (NDR) (Fig. 5d, e and Supplementary Fig. 4b). The NDR in *Atm*^*–/–*^ cells may be depleted of DNA owing to SPO11 double-cutting (see below).

### END-seq central peak reflects DNA-bound SPO11

At all hotspots, END-seq detected a uniform accumulation of reads aligned to the center of the DSB. This central peak was coincident with mapped SPO11 oligos, having a width (400 bp) similar to SPO11-oligo seq hotspots (300–400 bp) and was restricted to the NDR of H3K4/K36me3 (Fig. 2d,f and Supplementary Fig. 2b)^8^. Moreover, even low-level secondary oligos that are adjacent to the central SPO11 hotspot peak^8^ were detectable by END-seq (Fig. 2d). This suggested that the central peak represented a fraction of the total DSBs in the spermatocyte population in which SPO11 is not yet released, thereby highlighting the heterogeneity of cellular DSB processing.

**Fig. 6:**
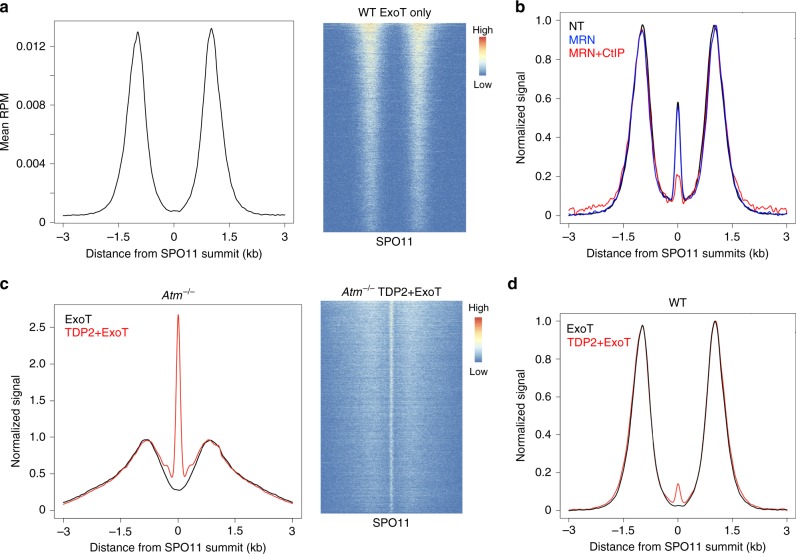
WT and *Atm*^*–/–*^ hotspots contain distinct species of DNA-bound SPO11. **a** END-seq processing with ExoT blunting alone shows total absence of SPO11 central peak when signal is aggregated around SPO11-oligo summits (left). Heatmap of ExoT only signal ± 3 kb around hotspot centers, ordered by total read count of END-seq (right). **b** Pretreatment with purified human MRN + CtIP reduces ExoVII + ExoT central peak detection (red) over no pretreatment (NT, black) and depends on the presence of CtIP (blue). Both MRN and MRN + CtIP reactions were carried out in the presence of manganese. One 12 dpp mouse used per condition. **c** END-seq with ExoT blunting alone also shows no central peak in *Atm*^–/–^ cells (black line). Pretreatment with purified human TDP2 before ExoT incubation recovers SPO11 signal, indicating abundance of unresected, bona fide SPO11 cleavage complexes (red line). Aggregate plot (left, normalized to same resection height) and heatmap (right) ±  3 kb around SPO11-oligo summits. **d** END-seq aggregate plot of WT ExoT blunting with (red line) and without (black line) TDP2 pretreatment shows only minor recovery of central peak signal.

If 5′ covalently bound SPO11 DSBs existed at the time of END-seq processing, then the proteinase K digestion would leave behind a two-bp 5′ overhang with a phosphotyrosyl bond that requires ExoVII digestion to fully blunt the end, analogous to our studies on TOP2 cleavage complexes (TOP2cc)^16^. To test this hypothesis, we performed END-seq with ExoT blunting only, allowing adapter ligation only to protein-free DNA ends while excluding ends with protein adducts, such as any remaining trapped SPO11 cleavage complexes (SPO11cc). Strikingly, ExoT detected only fully resected DNA ends with a total absence of central signal at all hotspots (Fig. 6a and Supplementary Fig. 4d). These data indicate that the central peak, which represents ~ 11% of the total DSB signal and is present all hotspots, reflects SPO11 covalently bound to its break site.

To validate that the ExoVII-dependent central peak was owing to SPO11 bound to the break and not some other form of occlusion, we speculated that it might be possible to remove SPO11 tyrosyl-linked DNA through incubation with purified human MRE11/RAD50/NBS1 (MRN) and CtIP prior to blunting with ExoVII + ExoT, mimicking the in vivo processing of meiotic DSBs. Indeed, we observed a dramatic loss of central signal by preincubation with MRN and CtIP (Fig. 6b)^39^. Incubation with MRN alone (prior to ExoVII + ExoT) or MRN and CtIP in the absence of manganese did not efficiently remove the central peak (Fig. 6b and Supplementary Fig. 4e) consistent with the finding that CtIP and manganese is required for the in vitro MRE11 endonuclease processing of protein-bound DSBs^40,41^. Thus, purified MRN + CtIP recognizes and removes the remaining SPO11 phosphotyrosyl bonds associated with the central signal. These data therefore support the idea that a fraction of SPO11 remains physiologically bound to a subset of breaks (~ 11%) at virtually all hotspots after cutting.

### Increased fraction of unresected SPO11-bound DSBs in *Atm*^–/–^

Because Tel1 regulates MRE11-initiated resection, there is a marked increase in unresected DSBs in Tel1-deficient yeast^6^. If ATM functions similarly to Tel1, then we would expect an accumulation of unresected SPO11cc, well above WT levels, at the center of the hotspots. The abundant MRE11 ChIP-seq signal specifically in *Atm*^–/–^ cells (Fig. 5c, d), indicative of incomplete processing, would also predict a vast increase in SPO11cc in the mutant.

We therefore sought to modify END-seq to specifically probe for SPO11cc. We hypothesized that preincubation with purified tyrosyl-DNA phosphodiesterase 2 (TDP2)^42^ would remove the remaining phosphotyrosyl adduct after proteinase K treatment, generating a two-nucleotide, protein-free 5′ overhang that ExoT could readily blunt for adapter ligation. Although ExoT alone detected no central peak in ATM-null cells, similar to WT (Fig. 6c), TDP2 + ExoT END-seq captured an astonishingly robust central signal (Fig. 6c). Moreover, this peak was strongly detected at all hotspots (Fig. 6c, right). SPO11cc detection by TDP2 + ExoT far exceeded the efficiency of detection with ExoVII + ExoT in *Atm*^–/–^ spermatocytes both at autosomes and at the non-PAR X chromosome (Supplementary Fig. 4f, g), most likely reflecting the biochemical preference for TDP2 over ExoVII to remove the phosphotyrosyl bonds and allow for adapter ligation^42^. These data indicate that ATM-deficient spermatocytes accumulate unresected SPO11cc, similar to yeast lacking Tel1^6^.

In WT cells, the ExoVII + ExoT central signal, which is also SPO11-bound, represents 11% of the total DSB fraction. Yet, in contrast to *Atm*^–/–^, combination of TDP2 and ExoT only recovered a small fraction (16%) of the ExoVII + ExoT central signal found in WT cells (Fig. 6d), corresponding to 0.5–2% of the total DSB signal. Given the high efficiency of TDP2 + ExoT in ATM-null cells for detection of SPO11cc, it was initially unclear why the WT TDP2 + ExoT central signal was so low. We speculated that in WT cells, the central signal might not simply reflect unresected SPO11cc, as observed in *Atm*^–/–^ spermatocytes. Rather, there might be an additional structure at the break site associated with SPO11-bound DNA in WT cells that somehow prevented recognition by TDP2.

### DNA-bound SPO11 is dependent on homolog engagement and PRDM9

**Fig. 7:**
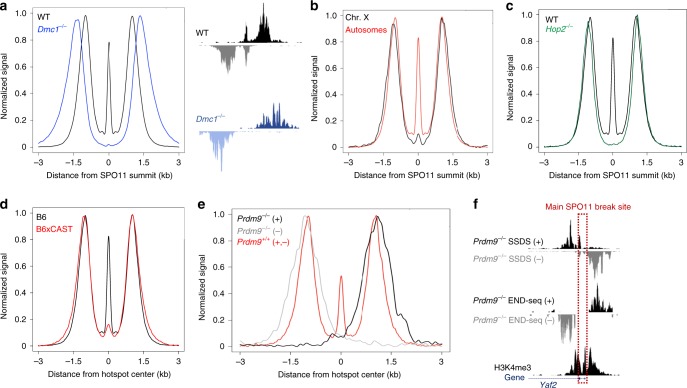
WT central peak relies on homolog engagement and PRDM9. **a** Aggregated END-seq signal around SPO11 oligos, with hotspot example, showing absence of SPO11 central peak in *Dmc1*^–/–^. **b** Reduction in central signal at non-PAR X chromosome hotspots; aggregated signal on ChrX compared with all autosomes. **c** Aggregated END-seq signal around SPO11 oligos showing absence of SPO11 central peak in *Hop2*^–/–^. **d** Aggregated END-seq signal of B6 (centered on B6 SPO11 oligos) versus B6xCAST hybrid (centered on hybrid SSDS hotspot centers). **e** *Prdm9*^*–/–*^ END-seq signal aggregated around default SSDS hotspot centers at ~ 200 sites with least overlap in SSDS top and bottom strands. WT END-seq is centered around WT SPO11 oligos at PRDM9-dependent hotspots. **f** *Prdm9*^*–/–*^ SSDS and END-seq tracks at a single default hotspot (*Yaf2* gene) with minimal SSDS top and bottom strand overlap. Main SPO11 break site (red dotted line) is inferred from SSDS pattern. Aggregate plots in all panels are normalized to the same height for visual comparison.

Our first clue to understanding the distinct nature of SPO11-bound DNA in WT vs. *Atm*^*–/–*^ cells was the observation that it was missing from all hotspots in *Dmc1*^*–/–*^ spermatocytes (Fig. 7a). This prompted us to ask whether the central peak associated with DNA-bound SPO11 was also dependent on ssDNA strand invasion into the homologous partner. Remarkably, we found a loss of central signal at all non-PAR X chromosome hotspots (Fig. 7b), which repair from the sister chromatid as the X chromosome has no homolog in males. The residual central signal resembled the TDP2 + ExoT signal in WT cells (Fig. 6d) and therefore might simply reflect the amount of unresected SPO11 naturally present on all chromosomes. In striking contrast, the central signal associated with autosomes and the non-PAR X chromosome was virtually identical in *Atm*^*–/–*^ spermatocytes, again confirming that the central signal is largely SPO11cc in the absence of ATM (Supplementary Fig. 4h–i). Finally, we found that the central signal was absent in *Hop2*^*−/−*^ spermatocytes (Fig. 7c), which fully load recombinases but fail to engage the homologous template^36^.

The analysis of hybrid mouse strains with different PRDM9 alleles has revealed that the degree of asymmetry in PRDM9 binding—that is, whether PRDM9 binds unequally to both homologs—predicts increased asynapsis and hybrid infertility^13,14^. When PRDM9 fails to bind the unbroken homologous chromatid, there is a severe reduction in both CO and NCO events^14,15^. Indeed, it has been suggested that similar to the X chromosome, asymmetric hotspots might be repaired from the sister chromatid^15^. We therefore performed END-seq on spermatocytes derived from juvenile B6xCAST hybrids with divergent genomes. Strikingly, the central peak was reduced to 2–3% of the total DSB signal in B6xCAST compared with the 11% observed in B6 (Fig. 7d) and much lower than in CAST alone (Supplementary Fig. 4j). Altogether, these findings support the idea that the central signal detected by ExoVII+ExoT in WT spermacotyes is associated with DNA-bound SPO11, and is dependent on the degree of homologous chromosome engagement.

In mice lacking PRDM9, DSBs occur at H3K4me3 sites mainly associated with promoters^9,11,12^. However, these DSBs are not repaired efficiently as COs resulting in meiotic arrest^11^. To test how PRDM9 deficiency impacts homolog engagement and resection, we performed END-seq on *Prdm9*^–/–^ spermatocytes. As SPO11 can generate multiple breaks within the H3K4me3 sites at promoters, we focused our analyses on *Prdm9*^–/–^ SSDS sites that exhibited the least overlap between top and bottom strand reads, i.e., hotspots that are most likely to have one main SPO11 cut site within the promoter (Supplementary Fig. 5a). Such SSDS hotspots showed limited signal at the center, in line with these sites having a strong preference for a single SPO11 cut site (Supplementary Fig. 5a, b). Strikingly, END-seq analysis of *Prdm9*^–/–^ spermatocytes revealed a total absence of the central peak at hotspots with the least SSDS strand overlap, examined either on aggregate or individually in the genome browser, whereas short- and long-range resection appeared to be relatively intact (Fig. 7e, f and Supplementary Fig. 5c, d). The absence of central signal is consistent with the idea that PRDM9 promotes homolog engagement, which in turn facilitates COs. Alternatively, delay in homolog engagement might allow more time for processing DNA-bound SPO11, resulting in loss of SPO11-bound DNA.

### SPO11 is associated with a recombination intermediate

**Fig. 8:**
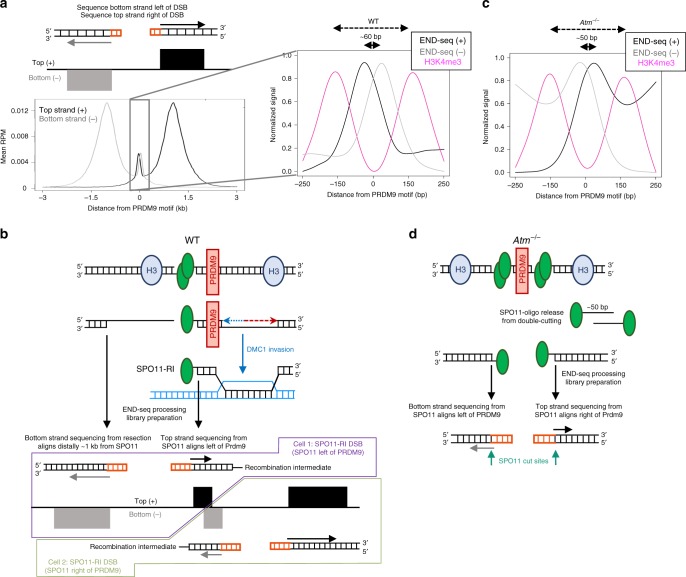
Polarity of END-seq reads reflects distinct natures of SPO11 cutting and processing in WT and ATM-null cells. **a** At fully processed and resected SPO11 DSBs, END-seq top and bottom strand reads exhibit a “correct” polarity to the right and left of the DSB, respectively (left). WT reads at the center of hotspots show a strand polarity that is reversed from what is expected (right, zoomed in at NDR). **b** PRDM9 as a barrier to SPO11 processing that results in a SPO11-bound recombination intermediate (SPO11-RI) structure. In WT, SPO11 cutting to one side of chromatin-bound PRDM9 within the NDR may block MRE11 activity on one side of the break, leaving SPO11 covalently bound to a short stretch of dsDNA, capping the DMC1-loaded ssDNA that extends ~ 1 kb from the NDR. SPO11-RI is sequenced starting from the first SPO11-bound nucleotide where an adapter is ligated. SPO11 that cut left of PRDM9 would result in top strand reads aligning to the left of the PRDM9 motif and bottom strand reads from fully resected ssDNA aligning at a distance away from the break site. **c** *Atm*^*–/–*^ END-seq central reads have correct separated strand polarity within the NDR. **d** Unresected SPO11 double-cutting within the same hotspot in *Atm*^*–/–*^ cells would show the correct polarity of top and bottom strands after adapter ligation to SPO11-bound DSBs and ~ 50 bp separation, as observed in **c**. Decreased MRE11 activity at these breaks would result in the direct sequencing of SPO11 cleavage complexes within the NDR rather than SPO11-RI in WT.

At a meiotic DSB that has been fully resected on both sides and SPO11 completely released, adapters ligated to the right end of the break will align to the top (+) DNA strand, whereas adapters ligated to the left end will align to the bottom (–) strand (Fig. 2c, Fig. 8a, left). When reads from all hotspots are aggregated, the distal resection signal around SPO11 cuts exhibited this “correct” polarity for the top and bottom strands (Fig. 8a, left). However, a close examination of the WT END-seq reads associated with the central signal unexpectedly revealed a “wrong” polarity, in which top strand reads aligned slightly to the left within the NDR and bottom strand reads aligned slightly to the right (Fig. 8a, right). As SPO11 generates a DSB with only 2 nt overhangs, if the central signal were merely a collection of unresected SPO11cc, then aggregating the top and bottom strand DSB endpoints should show no separation between them. However, we observed a top and bottom strand shift of ~ 60 nts in the “wrong” orientation (Fig. 8a, right). Detecting a significant reversal in the expected polarity indicated that the central peak was not simply SPO11cc, which should generate a canonical DSB pattern (see *Atm*^–/–^ below). This suggested that although SPO11 remains bound to a fraction DSBs, there is some kind of asymmetry associated with DNA-bound SPO11 that influenced END-seq detection compared with when SPO11 is released and both DNA ends are fully resected (Fig. 8a).

One potential mechanism that could contribute to the wrong polarity is asymmetric processing of SPO11. That is, if the two DNA ends bound by SPO11 are processed at different efficiencies by MRE11, one end might be incompletely processed, leaving SPO11 covalently bound to its cut site, whereas the other end is processed to completion and SPO11-oligo released. In this scenario, only the incompletely processed SPO11-bound end would contribute to central peak signal. The fully processed end (Fig. 8b, top, left end of the DSB) would result in the release of the SPO11-oligo that would generate a protein-free 3′ overhang. This (SPO11-free) DNA end would in turn be blunted by END-seq and sequencing reads would be detected within the distal, long-range resection peaks (Fig. 8b, bottom, left end of the DSB). In contrast, the other side of the DSB would be incompletely resected by MRE11 and retain SPO11 covalently bound to a two-nucleotide, 5′ overhang (Fig. 8b, top, right end of the DSB). END-seq detection (with ExoVII + ExoT) would then remove SPO11 and sequence the remaining dsDNA, with the first nucleotide sequenced being the SPO11 break site itself (Fig. 8b, bottom, right end of the DSB). This would result in top and bottom strand central peak reads with reversed polarity within the NDR, as SPO11 breaks to the left of the NDR center would contribute top strand reads aligning left of center, and SPO11 breaks to the right would contribute bottom strand reads aligning right of center (Fig. 8b). In a population of spermatoctyes in which both events occur among the cells, END-seq would detect an overall aggregated signal of resection reads with correct polarity and central reads with reversed polarity (Fig. 8b, bottom).

How could such asymmetric MRE11-mediated processing arise? Most SPO11-oligo-sequencing reads cluster in the center of the nucleosome-free depleted region where PRDM9 is also bound, suggesting that PRDM9 does not block SPO11 access^8,43^. Rather, we imagine that DNA-bound PRDM9 may guide the position at which SPO11 cuts within the nucleosome-free region, which might be slightly displaced on average by 30 base-pairs (half the size of 60 bp shift) from PRDM9 itself (Fig. 8b). It has been suggested that PRDM9 often remains bound on the uncut chromosome^14^, whereas SPO11 has been proposed to be associated with DNA ends^19^ until or even subsequent to strand invasion^14,19^. If PRDM9 similarly remains bound to DNA post-cleavage between the SPO11 cut and MRE11-endonucleolytic nicking position (Fig. 8b), it could interfere with MRE11 release of SPO11. This would prevent MRE11 from generating a fully ssDNA overhang only on one side of the DSB (Fig. 8b, top). Because the natural on/off binding affinity of PRDM9 would determine the frequency at which MRE11 activity is blocked and SPO11-bound DNA is captured by END-seq, we would expect that the central peak to be detected at all hotspots genome-wide, as observed (Fig. 2b). Moreover, all hotspots had equal ratios of central peak to resection signal (~ 11%), indicating that no hotspot had preference over others, regardless of break frequency.

The central signal not only reflected asymmetric MRE11-mediated processing, but also required DMC1- and HOP2-mediated strand invasion and engagement with the homologous chromosome template (as shown above). Owing to this dependency, we infer that SPO11 remains bound post homolog engagement and during the formation of a recombination intermediate (RI). We therefore refer to this RI, with SPO11 capping the 3′ resected end, as SPO11-RI (Fig. 8b). Consistent with our results, an independent genomic sequencing method (S1-seq) corroborates the presence of SPO11-RI with a reversed central signal polarity in WT mouse spermatocytes^38^.

### Increased SPO11 double-cutting at the same hotspot in *Atm*^–/–^

The central signal in ATM-null spermatocytes is largely comprised of unresected SPO11cc, which accumulates MRE11 in vivo and is sensitive to TDP2-mediated processing (Figs. 5d, 6c, and Supplementary Fig. 4f–i). This is distinct from the enrichment of SPO11-RI observed in WT cells. Elevated levels of SPO11cc in *Atm*^–/–^ cells could arise from decreased MRE11 endonucleolytic or 3′−5′ exonuclease activity (Fig. 5c, d), which is also characteristic of yeast *Tel1* deficiency^6^. If *Atm*^–/–^ SPO11cc reflects fully unresected DSBs and not SPO11-RI, then the aggregated END-seq signal should have no obvious polarity. To examine this, we strand separated the central signal in *Atm*^–/–^ cells. Unexpectedly, the separated strands exhibited a ~50 base-pair gap within the nucleosome-depleted region with the correct DSB polarity (Fig. 8c). If SPO11 cut once on each chromatid throughout the NDR within the population of cells and remained bound to DNA, there would be a 2-nt gap between top and bottom strand DSB endpoints. We therefore infer that the larger gap size reflects frequent SPO11 double-cutting within the same hotspot (Fig. 8c, d)^44^. These measurements are consistent with the increased 40–70 nucleotide SPO11-oligo species that were detected in ATM-null mice^7^, as two distinct SPO11 cuts adjacent to one another could release these longer oligos even without MRE11 endonuclease activity (Fig. 8d). Moreover, MRE11 ChIP-seq revealed a notable dip in MRE11-binding exactly within the NDR (Fig. 5d, e and Supplementary Fig. 4b), consistent with the loss of DNA within hotspot centers. These results are also supported by genetic evidence of double-cutting in *Atm*^*–/–*^ spermatocytes (A. Lukaszewicz, S. Keeney and M. Jasin, personal communication).

Increased double-cutting around PRDM9-binding sites would preclude it from blocking any MRE11 short-range resection that does occur, thereby reducing the frequency of SPO11-RIs in ATM-null cells (Fig. 8d). Our finding that central signal in *Atm*^*–/–*^ spermatocytes exhibits the correct polarity (Fig. 8b), and that this signal is identical on the autosomes and non-PAR X chromosome (Supplementary Fig. 4h, i), is consistent with a significant reduction in SPO11-RI in *Atm*^*–/–*^ spermatocytes. Therefore, through its regulation of SPO11 cutting and resection, ATM indirectly regulates the formation of SPO11-RI.

## Discussion

We show here the capacity of END-seq to elucidate early meiotic pathways that are critical for proper chromosome segregation and fertility. Because the method requires little starting material (as low as a single mouse) and can be modified through differential enzymatic reactions to detect distinct DSB structures, we are able to bypass the limitations imposed by previous meiotic hotspot profiling techniques. In doing so, we uncovered a pattern of resection, strikingly uniform at all DSBs. This reflects the fact that short- and long-range resection are tightly coupled in a single processive reaction that is mediated by ATM. This pattern is highly reminiscent of meiotic resection in yeast, and reflects the evolutionary conservation of DSB processing pathways.

Although yeast do not possess obvious homologs of BRCA1, BRCA1 has a well-described function in supporting the resection of DSBs in somatic mammalian cells^32^. Surprisingly, we find that BRCA1 does not similarly promote resection during meiotic recombination. One potential reason could be that 53BP1 is not recruited to DSBs in mitotic cells or in early prophase meiocytes^45,46^, which may reflect similarities in pathways that suppress DSB repair during mitosis and meiosis. If the primary function of BRCA1 is to counteract 53BP1’s block to resection at DSBs, this function of BRCA1 would not be needed during mitosis or meiosis.

In addition to promoting resection in somatic cells, BRCA1 also facilitates the loading of RAD51/DMC1 onto ssDNA^47^, a function which appears to be conserved in meiosis^35^. In several BRCA1-deficient mouse strains with known defects in RAD51/DMC1 filament formation, we observed a slight increase in resection tracts. We suggest that this reflects an indirect role for BRCA1 in limiting resection by promoting recombinase loading onto ssDNA. Consistent with this idea, *Dmc1*^–/–^ but not recombinase-proficient *Hop2*^–/–^ spermatocytes displayed a marked increase in minimum and maximum resection endpoints. We also observed hyper-resection in ATM-null spermatocytes, which we propose is owing in part to the limited availability of recombinases to form filaments in the prescence of excessive SPO11 cutting. Thus, we imagine that in BRCA1−, DMC1−, and ATM-deficient spermatocytes, defective recombinase loading permits reiterative engagement of the long-range resection machinery.

Although some aspects of resection are highly conserved in yeast and mammals, SPO11 processing appears not to be. Unlike yeast, mammalian cells accumulate significant levels of DNA-bound SPO11 that represent SPO11-RI. Our model suggests that incomplete processing of SPO11 in mammals, perhaps owing to PRDM9 blocking, still allows for, and may even promote, strand invasion via DMC1 into a homologous template (Fig. 8b). Although SPO11-RI is readily detectable by END-seq using ExoVII + ExoT and represents 11% of the total DSB signal, processing with TDP2 + ExoT only detects SPO11cc (Fig. 6d). We therefore estimate that out of the total DSB signal detected by ExoVII + ExoT in WT cells, 0.5–2% is truly unresected SPO11cc, as detected by TDP2 + ExoT, and the remaining ~ 10% is SPO11-RI.

What is the biological relevance of SPO11-RI? One possibility is that SPO11-RI facilitates NCO and/or CO events necessary for the exchange of genetic information between homologs. The majority of NCO and CO events occur only in the 4000 hottest hotspots^15^, and we have found that SPO11-RI occurs across all 5000 DSBs detected by END-seq (Fig. 2b). NCO and CO events are reduced in strains with asymmetric PRDM9 hotspots, and we found strong depletion of the central signal in hybrid strains. CO were less likely to occur in gene-rich regions^15^, and we have found that the central signal is decreased with increasing gene expression independent of DSB formation (Supplementary Fig. 5e, f). Finally, the positioning of NCO and CO tracts are highly enriched in a small region (generally <500 bp) surrounding the PRDM9 motifs^15,21^. It is possible that the “capping” of the 3′ ssDNA by SPO11 (Fig. 8b) delays polymerization or double Holliday junction migration, therefore restricting SPO11-RI to a 400 bp region surrounding the PRDM9 motif. Although SPO11-RI correlates with NCO and CO events, additional studies will be necessary to determine its precise physiological function.

The loss of the central signal in mouse hybrids, in *Dmc1*^*–/–*^ mice, and on the WT non-PAR X chromosome could potentially be explained if DSB repair is delayed. With a fixed on/off rate for PRDM9, the longer DSBs persist, the more time MRE11 may have to process and remove SPO11-bound DNA, as PRDM9 will eventually exit the hotspot. Similarly, in PRDM9-deficient mice with defective repair, DSBs persist longer, and SPO11-RI is absent.

We find limited evidence of SPO11-RI in ATM-null cells. Instead there is a concomitant increase in SPO11cc and MRE11 binding owing to incompletely processed DSBs. Recent studies have demonstrated that some of these DSBs arising in *Atm*^–/–^ mice can be repaired by detrimental non homologous end-joining (NHEJ) (A. Lukaszewicz, S. Keeney and M. Jasin, personal communication). Given the marked in vitro activity of TDP2 in *Atm*^–/–^ spermatocytes, SPO11cc might be rejoined in part by TDP2-dependent NHEJ in vivo. Direct hydrolysis of PRDM9-blocked SPO11 by TDP2 in WT cells would also likely result in aberrant end-joining. However, we found that purified TDP2 does not act on SPO11-RI in vitro (Fig. 6d). Although it remains unclear why SPO11-RI is an inefficient substrate for TDP2, it is possible that TDP2 cannot recognize SPO11-RI owing to steric hindrance associated with the heteroduplex DNA. If TDP2 were similarly inactive on SPO11-RI in vivo, the formation of SPO11-RI could serve to prevent NHEJ and instead favor repair towards high-fidelity HR.

In conclusion, both ATM and PRDM9 orchestrate mammalian SPO11 processing in a manner that influences meiotic DSB repair.

## Methods

### Mice

C57BL/6 mice (Jackson Laboratory) were bred in-house, and male pups were killed at 12–14 dpp for testes isolation. *Atm*^–/–^ mice (Barlow et al. 1996) were backcrossed eleven generations to C57BL/6 mice. *Exo1*^–/–^ mice were a gift from Winfried Edelmann. *Dmc1*^–/–^ mice were a gift from Scott Keeney. *53bp1*^–/–^ mice were a gift from Junjie Chen. *Prdm9*^–/–^ mice were a gift from Petko Petkov. *Brca1*^*+/△11*^ mice were obtained from the NCI mouse repository. *53bp1*^*S25A*^ mice were generated as described^33^. *p53*^–/–^ mice were purchased from Taconic Biosciences. Strains were crossed to generate *Brca1*^*Δ11*^*53bp1*^–/–^, *Brca1*^*△11*^*53bp1*^*S25A*^ and *Brca1*^*△11*^*p53*^*+/–*^ mice. CAST/EiJ (males) were purchased from Jackson Laboratory and were crossed with C57BL/6 (females) for one generation to produce B6xCAST F1 hybrids, respectively. All mouse breeding and experimentation followed protocols approved by the National Institutes of Health Institutional Animal Care and Use Committee.

### Mouse testicular cell isolation

Adapted from Baker et al. 2014. Testes were dissected from 12–14 dpp male mice and placed into a 6 cm tissue culture dish containing Dulbecco's Modified Eagle Medium (DMEM). Tunica albuginea were removed under a microscope, and tubules were gently dissociated with forceps and placed into 50 mL tube containing 20 mL DMEM. After tubules settled to the bottom of the tube, DMEM was aspirated and replaced with 20 mL DMEM containing 0.5 mg/mL Liberase TM (Roche, 5401127001) and incubated at 32 °C for 15 min at 500 rpm. Tubules were washed once with fresh DMEM, replaced with 20 mL DMEM containing 0.5 mg/mL Liberase TM and 100 U DNase I (ThermoFisher, EN0521), and incubated at 32 °C for 15 min at 500 rpm.

Tubules were disrupted by gentle pipetting and passed through a 70-μm Nylon cell strainer (Falcon) repeatedly until tissue debris was fully removed. Cells were pelleted at 1500 rpm at 4 °C for 5 min and washed with 10 mL DMEM. Cells were filtered through a 40 μm Nylon cell strainer (Falcon) repeatedly until debris was fully removed and pelleted at 1500 rpm at 4 °C for 5 min. Cells were resuspended in 1 mL phosphate-buffered saline (PBS) and counted.

### END-seq

Single-cell suspensions of bulk testicular cells were immediately embedded after isolation into 0.75% agarose plugs. After isolation, cells in 1 mL PBS were diluted to 5–7 million bulk cells/mL PBS and separated into 1 mL of cells per 1.5 mL tube for plug making. Spike-in cells were added at 2% of bulk cell number per tube/plug. Multiple plugs were made per sample if necessary, depending on number of mice and total cell number isolated, processed in the same tube, and DNA later combined after plug melting.

A detailed description for embedding cells into agarose plugs and general END-seq procedure can be found in Canela et al.^16^, Canela et al.^17^. In brief, agarose embedded cells were solidified at 4 °C for 15 min then immediately lysed and digested with Proteinase K at 50 °C for 1 hr then 37 °C for 7 hr. Plugs were then washed with TE, treated with RNase at 37 °C for 1 h, and stored at 4 °C for no longer than 1 week before the next series of enzymatic reactions.

Unless otherwise noted, plugs were treated with sequential combination of Exonuclease VII (NEB) for 1 h at 37 °C followed by Exonuclease T (NEB) for 45 min at 24 °C to blunt DNA ends before Illumina adapter ligation^16^. For experiments in which only Exonuclease T was used to blunt, plugs were digested with Exonuclease T for 1 h at 24 °C. For experiments with purified human TDP2 (gift from Keith W. Caldecott), plugs were treated with 500 pm TDP2 (in 50 mm Tris-HCl pH 8.0, 10 mm MgCl_2_, 80 mm KCl, 1 mm DTT, and 0.05% Tween−20) for 4 h at 24 °C, followed by Exonuclease T digestion for 1 h at 24 °C.

For experiments with purified human MRE11/RAD50/NBS1 (MRN) and CtIP (Tanya Paull lab), plugs were treated with 50 nm MRN (MR and NBS1 were pre-incubated for 10 min at 4 °C prior to reactions) with or without 80 nm CtIP (in 25 mm MOPS pH 7.0, 20 mm Tris-HCl pH 8.0, 80 mm NaCl, 1 mm DTT, 1 mm ATP, 5 mm MgCl_2_, 1 mm MnCl_2,_ and 0.2 mg/mL bovine serum albumin) for 1.5 h at 37 °C. Plugs were treated again with proteinase K for 1 h at 50 °C to degrade any remaining bound protein, followed by Exonuclease VII and Exonuclease T digestion as previously described.

For all enzymatic reactions, subsequent steps of A-tailing, adapter ligation, plug melting, chromatin shearing, and second round of adapter ligation for sequencing were performed exactly as previously described^16,17^.

### ChIP-seq

Ten million bulk testicular cells were fixed in 1% formaldehyde (Sigma, F1635) at 37 °C for 10 min. Fixation was quenched with glycine (Sigma) at a final concentration of 125 mm. Cells were washed twice with cold PBS, and pellets were snap frozen on dry ice and stored at −80 °C until sonication. Sonication, immunoprecipation, and library preparation were performed as previously described in (Canela et al.^16^). In brief, frozen pellets were resuspended in 1 mL cold RIPA buffer (10 mm Tris-HCl pH 7.5, 1 mm ethylenediaminetetraacetic acid (EDTA), 0.1% sodium dodecyl sulphate, 0.1% sodium deoxycholate, 1% Triton X-100, and 1 Complete Mini EDTA-free proteinase inhibitor tablet (Roche)) and sonicated using a Covaris S220 at duty cycle 20%, peak incident power 175, and cycle/burst 200 for 30 min at 4 °C.

Chromatin was precleared with 40 μL prewashed Dynabeads Protein A (ThermoFisher) for 30 min at 4 °C, followed by incubation with 40 μL Dynabeads Protein A bound to either 6 μL anti-H3K4me3 (Millipore, 07–473) or 4 μL anti-MRE11 (Novus, NB100-142) overnight at 4 °C. Beads were washed and cross-linking reversed the next day as described in (Canela et al.^16^). Immunoprecipitated DNA was removed from beads and stored at −20 °C until library preparation, which was performed as described in (Canela et al.^16^).

### END-seq data analysis

Reads were aligned to the mouse (GRCm38p2/mm10) genome using Bowtie version 1.1.2^48^ and three mismatches were allowed and the best strata for reads were kept with multiple alignments (-n 3 -k 1 -l 50). Functions “view” and “sort” of samtools (version 1.6)^49,50^ were used to convert and sort the mapping output to sorted bam file. Peaks were called using MACS 1.4.3^51^. As the strand separated aggregate plots (Fig. 2c) show ~ 2 kb distance, END-seq peaks were called using the parameters:–shiftsize = 1000, –nolambda, –nomodel, and–keep-dup = all. Peaks with >2.5-fold-enrichment are kept and those within blacklisted regions (https://sites.google.com/site/anshulkundaje/projects/blacklists) were filtered.

For estimation of total breaks and comparison between genotypes, we added a spike-in control into END-seq samples that consists of a G1-arrested Ableson-transformed pre-B cell line (*Lig4*^*−/−*^) carrying a single zinc-finger-induced DSB at the TCRβ enhancer. This site is expected to break in all spike-in cells, which were mixed in at a 2% frequency with bulk testicular cells. END-seq signal was calculated, as RPKM, within ± 5 kb window around all hotspot centers. Total intensity was divided by the signal around the spiked-in breaks and then divided by 50 since the spiked-in was added at a 1:50 ratio (2%).

As the pattern of END-seq peaks are consisted of continuously sharp peaks and it is quite reproducible between experiments and to estimate the distance between the shar peaks and study its relation with nucleosome positioning, END-seq peaks were split as subpeaks by PeakSplitter tool of PeakAnalyzer with default parameters (https://www.bioinformatics.org/peakanalyzer/wiki/Main/Overview^52^) and the distances of sharp peak summits within each break were calculated.

### Signal-to-noise analysis

ENCODE measures Signal-to-noise(S/N) ratio by fraction of reads in peaks (FRiP) and cross-correlation profiles (CCPs) for ChIP-seq. We used it for END-seq here.

The FRiP value is fractions of reads that mapped into called peaks without any filtering. The higher FRiP, the more enrichment. ENCODE recommends the threshold for FRiP is more than 1% for ChIP-seq.

The CCPs assess the quality of END-seq enrichment over background independent of peak calling. The plot shows the Pearson cross-correlations (CCs, *y* axis) of reads intensities between the plus strand and the minus strand, after shifting minus strand (*x* axis). There are two peaks, one corresponding to read length (CCread, blue dash line) and the other one corresponding to the fragment length (CCfrag, red dash line). Normalized strand coefficient (NSC) is CCfrag divided by minimal CC value (CCmin) and relative strand coefficient (RSC) is the ratio of CCfrag-CCmin divided by CCread-CCmin. The higher NSC and RSC, the more enrichment. ENCODE recommends an NSC ≥ 1.5 and RSC ≥ 0.8 for ChIP-seq.

The CCPs profile, NSC value and RSC value were generated by Phantompeakqualtools (https://github.com/kundajelab/phantompeakqualtools^22,53^). Different to ChIP-seq pattern that plus strand signal is at the left of minus strand signal, END-seq has opposite direction, so the shiftsize of the fragment is negative. Unsurprising, the absolute shift values are consistent with the distance of two long-range resection peaks showed by the aggregate plots.

### Quantification of resections

Short-range resection was measured by a sliding window containing 10 bp bins, starting from the peak of long-range resection signal into the hotspot center. Mean value of ± 500 bp window around the peak site was used to estimate the normal distribution of the resection endpoints. Comparing the real signal of the sliding window to it, when the window got *p* value ≤ 0.05 by one-sample *t* test (alternative = “less”), the distance from hotspot center to the window midpoint was defined as the short-range resection length. Plus strand and minus strand were calculated separately.

For each hotspot, the long-range resection describes the distribution of resection endpoints among the cell population. Mean long-range resection was quantified based on the definition of expect value of a distribution as:

$$E\left( x \right) = \mathop {\sum}\limits_{{\it{n}} = 21}^{400} {\left( {x_ip_i} \right)}$$

RPM were calculated for 10-bp bins in a ± 4 kb window of each SPO11 summit. The first bins were not considered, the calculation started from the 21th bin. *x*_*i*_ is distance of the current bin to SPO11 summit and *p*_*i*_ is the probabilities calculated as RPM of the current bin/total RPM from 21th to 400th bins. Plus strand and minus strand were calculated separately. Top 250 breaks were used for the boxplot of all mean resection comparison.

To measure maximum long-range resection, a sliding window containing 20-bp bins was used, starting from the peak of long-range resection to further region until more than half of bins within current window had lower intensity than background. The location of the last bin with a detectable signal over background is regarded as the maximum resection endpoint. Dynamic background was determined by the maximum intensity of 20-bp bins within 5 ~ 6 kb region away from SPO11 summit for individual hotspot. Plus strand and minus strand were calculated separately.

### Estimation of SPO11-RI and unresected SPO11cc

The fraction of central signal detected by ExoVII + ExoT or TDP2 + ExoT in B6 was calculated using the total area substrate the area of ExoT blunting only after forcing them to the same height and then divided by ExoVII + ExoT total area. We got 11% for ExoVII + ExoT and 1.7% for TDP2 + ExoT. As the central signal of ExoVII + ExoT consists of both SPO11-RI and unresected SPO11cc, we used TDP2 to measure fraction of the unresected SPO11cc. The detection ability of ExoT for the small overhangs of SPO11cc and long overhangs of SPO11-RI may differ. Assuming ExoT detected unresected SPO11cc and SPO11-RI at the same level, the unresected SPO11cc fraction will be 1.7%. However, ExoT might work better for unresected SPO11cc detection than SPO11-RI and assume it captures all unresected SPO11cc. We first quantified the total break number for ExoT blunting sample using spike-in normalization which is 93. Then multiplied the ratio of the central signal with TDP2 + ExoT vs ExoT only (~2%). The number of unresected SPO11cc is ~ 2 and is 0.6% of total break number. Thus, the fraction of unresected SPO11cc could be 0.6% ~ 1.7% and SPO11-RI is 9.3% ~ 10.4%.

### Mouse hybrid END-seq analysis

Single-nucleotide polymorphisms (SNP) were annotated for CAST genome by the Sanger Mouse Genome Project^54^ based on B6 mouse reference genome (mm10). Only SNPs (*n* = 20,668,274) passed all filter criteria were employed to generate an “N-masked” genome. In all, 14,951 hotspots in B6xCAST hybrid genome were download from GSE75419. As Bowtie does not allow gapped alignment for Ns, the hybrid END-seq reads were aligned by Bowtie2 version 2.3.5.1^55^ with options -N 1 -k 1.

### SPO11 oligos, SSDS, and ChIP-seq and RNA-seq data analysis

For SPO11-oligo seq, reads were aligned to the mouse (GRCm38p2/mm10) genome using Bowtie version 1.1.2 with options -n 2 -m 1 -l 50. Peaks were called using MACS 1.4.3. Peaks were called using the parameters: –shiftsize = 73,–nomodel and then filtered by fold-enrichment (FC ≥ 10) as well as the blacklists. SPO11 summits from SPO11-oligo seq overlapped the B6 END-seq peaks (~ 5000) were used for most of the aggregate plots except the B6xCAST hybrid data. For DMC1 SSDS, type1 alignments and hotspots’ coordinates of SSDS data were directly download from GEO database. B6xCAST hybrid SSDS was analyzed as described in END-seq part. For our H3K4me3 ChIP-seq and the public H3K36me3 ChIP-seq, reads were aligned to the mouse (GRCm38p2/mm10) genome using Bowtie version 1.1.2 with options -n 2 -m 1 -l 50. For RNA-seq, reads were aligned to the mouse (GRCm38p2/mm10) genome using TopHat version 2.1.1 (PMID: 19289445) with default parameters. The gene expression level of RPKM was determined by Cuffnorm version 2.2.1^56^ based on the annotation from RefSeq. Mean values across samples were used.

### Data visualization

Aligned-reads bed files were first converted to bedgraph files using bedtools genomecov (PMID: 20110278) following by bedGraphToBigWig to make a bigwig file^57^. Visualization of genomic profiles was done by the UCSC browser^58^. Genome browser profiles were normalized to the library size (RPM). Heatmaps were produced using the R package ‘pheatmap’.

Aggregate plots for sequencing data around hotspot centers or SPO11 summits were performed as follows: a fixed window was defined around all sites genome wide. The number of reads overlapping each nucleotide was calculated. The aggregate signal was smoothed using smooth.spline function in R. For END-seq, only the adapter ligated endpoints were used.

### Statistical analysis

Statistical analysis was performed using R version 3.5.0 (http://www.r-project.org). The statistical tests are reported in the figure legend and main text.

### Ethical compliance

All mouse breeding and experimentation followed protocols approved by the National Institutes of Health Institutional Animal Care and Use Committee.

### Reporting summary

Further information on research design is available in the Nature Research Reporting Summary linked to this article.

## Supplementary information

Supplementary Information

Reporting Summary

Description of Additional Supplementary Files

Supplementary Data 1

Supplementary Data 2

## Data Availability

The data that support the findings of this study (END-seq and ChIP-seq) are available in the GEO under accession GSE138915. RNA-seq data were downloaded from GEO accession GSE61613^59^. H3K36me3 ChIP-seq was downloaded from GEO accession GSE76416^60^. Prdm9^−/−^ DMC1 SSDS were downloaded from GEO accession GSE99921^24^. DMC1 SSDS in the B6xCAST hybrid, B6 and CAST were downloaded from GEO accession GSE75419^20^. SPO11-oligo sequencing data were downloaded from GEO accession GSE84689^8^. The source data underlying Supplementary Data 1 (Peaks called from END-seq in WT mice) and Supplementary Data 2 (Peaks called from END-seq in *Atm*^*−/−*^ mice) are provided as a Supplementary Data files. Any additional data are available on request from the authors.

## Code availability

R code used for analysis is available at: https://github.com/weiwsmiling/meiosis_END-seq
