# Supplementary material for: ATM and PRDM9 regulate SPO11-bound recombination intermediates during meiosis
Source: Nat Commun. 2020 Feb 12;11:857. doi: 10.1038/s41467-020-14654-w (PMC7016097; doi:10.1038/s41467-020-14654-w)
Supplement: Supplementary file 1 — Supplementary Information [file 41467_2020_14654_MOESM1_ESM.pdf]

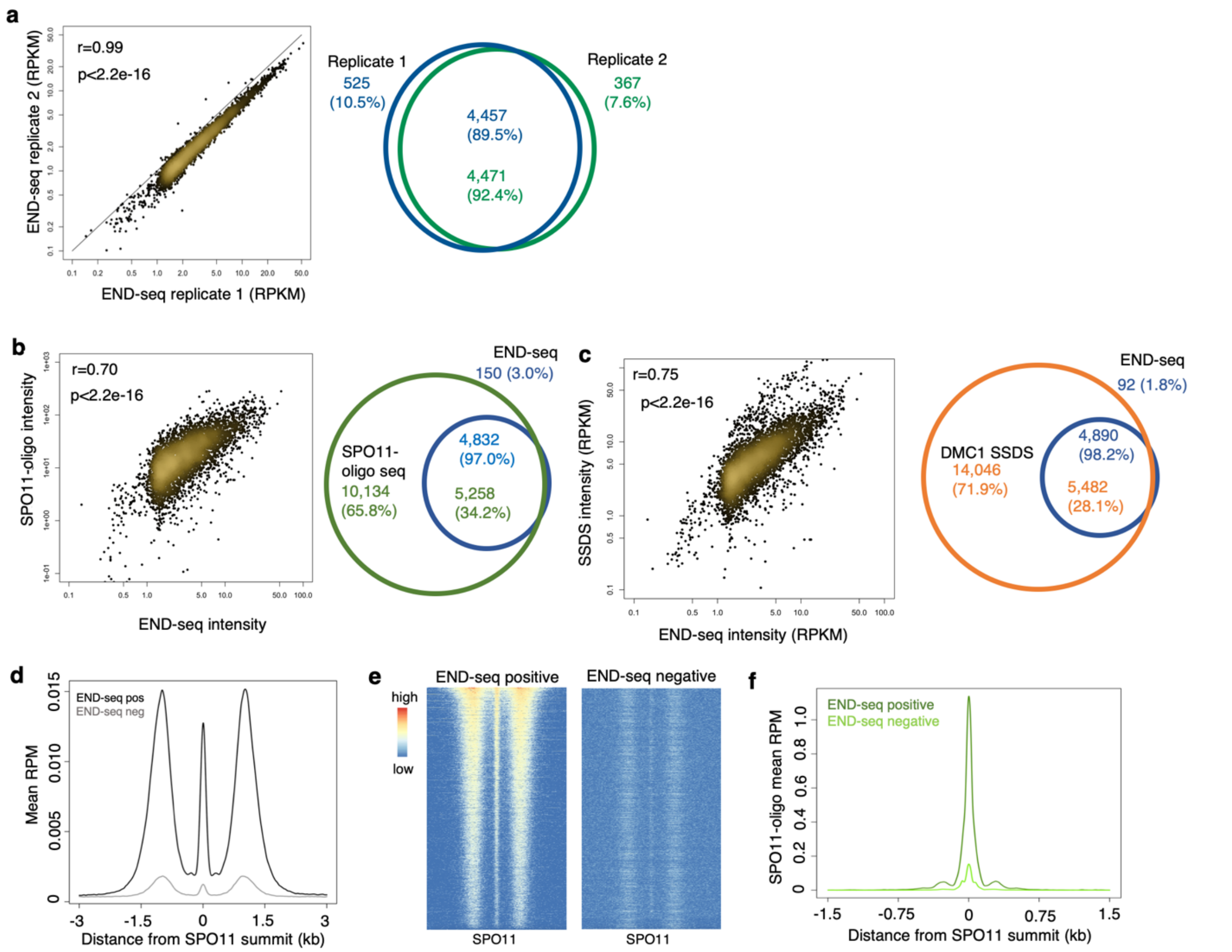

Supplementary Fig. 1 Assessing END-seq accuracy and sensitivity. (**a**) Comparison of two WT END-seq biological replicates each made from 20 pooled juvenile mice. Left panel: Correlation (Pearson's r) between break intensity of the two replicates in a ±3kb window around SPO11 summits. Right panel: Venn diagram showing overlap of peak calling from two END-seq replicates. P value <2.2e-16, fisher's exact test. (**b**) Comparison of END-seq and SPO11-oligo sequencing hotspot mapping. Left panel: Correlation (Spearman's r) of END-seq and SPO11-oligo intensity in a ±3kb window around SPO11 summits. Right panel: Venn diagram showing overlap of END-seq peaks and SPO11-oligo peaks. P value <2.2e-16, fisher's exact test. (**c**) Comparison of END-seq and DMC1 SSDS hotspot mapping. Left panel: Correlation (Spearman's r) of END-seq and SSDS intensity in a ±3kb window around SPO11 summits. Right panel: Venn diagram shows overlap of END-seq peaks and SSDS peaks. P value <2.2e-16, fisher's exact test. (**d**) Aggregated END-seq signal at SPO11-oligo sites for END-seq peak called hotspots (END-seq positive) versus non-peak called hotspots (END-seq negative) around hotspot centers. (**e**) Heatmaps of END-seq signal for END-seq positive or negative hotspots ±2.5kb around SPO11 summits. (**f**) Aggregated SPO11-oligo seq signal at END-seq positive versus END-seq negative hotspots around SPO11 summits.

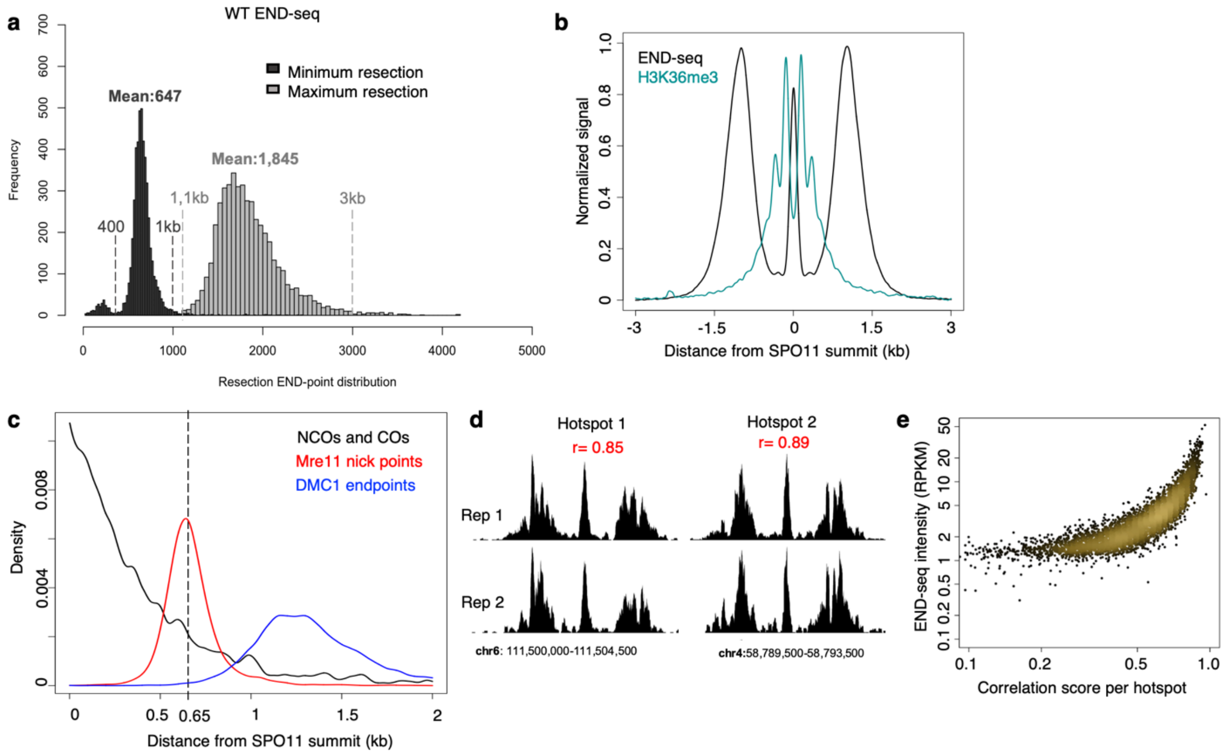

Supplementary Fig. 2 Resection calculations and reproducibility between hotspots. (**a**) Histogram distributions of END-seq minimum resection lengths and maximum long-range resection endpoints in WT spermatocytes. Mean values (bp) are listed. (**b**) Aggregate signal of WT END-seq and WT H3K36me3 ChIP-seq around SPO11-oligo summits at top 5000 breaks. Signals are normalized to the same height. (**c**) Distribution of short-range resection endpoints by END-seq (red), crossover breakpoints and noncrossover midpoints (black), and DMC1 resection endpoints (blue) relative to SPO11-oligo summits. (**d**) Between two biological END-seq replicates, hotspot break pattern is highly reproducible. Hotspots were binned into 20nt bins and reads per bin were correlated between replicates (Pearson correlation). Two hotspot examples demonstrate the reproducibility in resection pattern. (**e**) Correlation score (determined in "**d**") plotted against END-seq intensity per hotspot. Hotter hotspots produce more reproducible resection patterns between replicates.

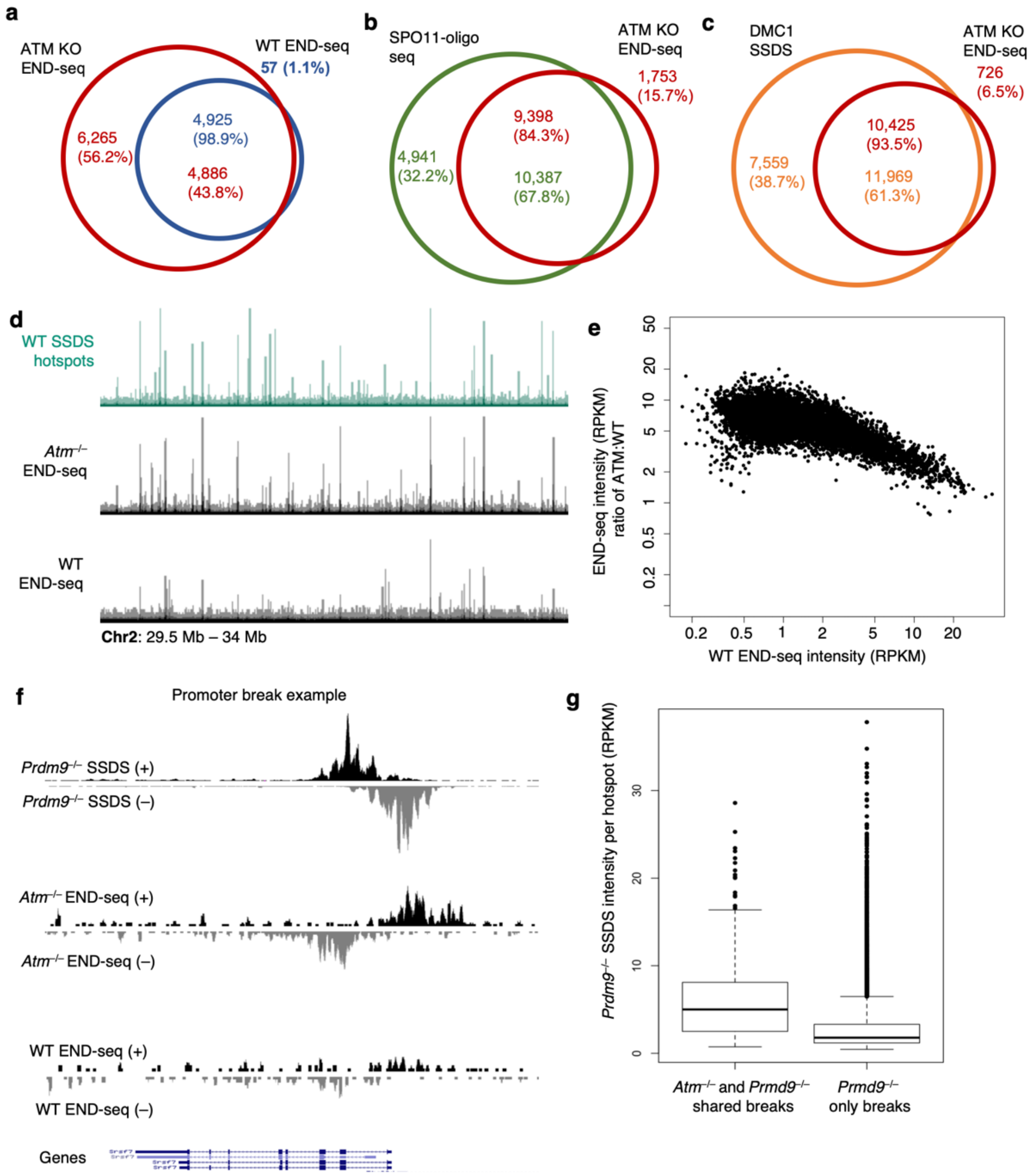

Supplementary Fig. 3 Comparing ATM-null END-seq to previous hotspot mapping methods. (**a**) Comparison of WT and *Atm*$^{-/-}$ END-seq. Venn diagram shows overlap between WT and *Atm*$^{-/-}$ END-seq peaks. P value <2.2e-16, fisher's exact test. *Atm*$^{-/-}$ END-seq peaks are provided in Supplementary Table 2. (**b**) Venn diagram shows overlap of *Atm*$^{-/-}$ END-seq peaks and B6 SPO11-oligo sequencing peaks. P value <2.2e-16, fisher's exact test. Of the 1,753 hotspots found in ATM-null END-seq and not SPO11-oligo seq, 48% (833 out of 1,753) overlap with breaks specific to *Prdm9*$^{-/-}$ SSDS hotspots, of which 48% are at promoters. (**c**) Venn diagram shows overlap of *Atm*$^{-/-}$ END-seq peaks and DMC1 SSDS peaks. P value <2.2e-16, fisher's exact test. Of the 726 hotspots found in ATM-null END-seq and not SSDS, 82% (597 out of 726) overlap with breaks specific to *Prdm9*$^{-/-}$ SSDS hotspots, of which 59% are at promoters. (**d**) Representative genome browser profiles of meiotic hotspots on Chromosome 2 for WT SSDS, *Atm*$^{-/-}$ END-seq, and WT END-seq. Browser axis scales are equal for *Atm*$^{-/-}$ and WT END-seq tracks to highlight the increased signal in the absence of ATM at typically cold hotspots. (**e**) The END-seq intensity of weaker hotspots preferentially increase more than

stronger hotspots in *Atm*$^{-/-}$ mice. WT RPKM per hotspot plotted against RPKM ratio of *Atm*$^{-/-}$ to WT (signal normalized to spike-in control). (**f**) Genome browser example of an ATM-null END-seq break at a *Prdm9*$^{-/-}$ SSDS promoter hotspot. Top (+) and bottom (–) strand-separated *Prdm9*$^{-/-}$ SSDS and *Atm*$^{-/-}$ END-seq tracks show significant signal at the promoter of the Srsf7 gene (bottom). WT END-seq tracks are shown to the same scale as ATM-null, illustrating that these breaks are specific to the loss of ATM regulation of SPO11. (**g**) Boxplot comparison of *Prdm9*$^{-/-}$ SSDS intensity per hotspot of *Atm*$^{-/-}$ and *Prdm9*$^{-/-}$ shared hotspots versus hotspots found only in *Prdm9*$^{-/-}$ mice. Hotspots shared between *Atm*$^{-/-}$ END-seq and *Prdm9*$^{-/-}$ SSDS are among the hottest *Prdm9*$^{-/-}$ hotspots.

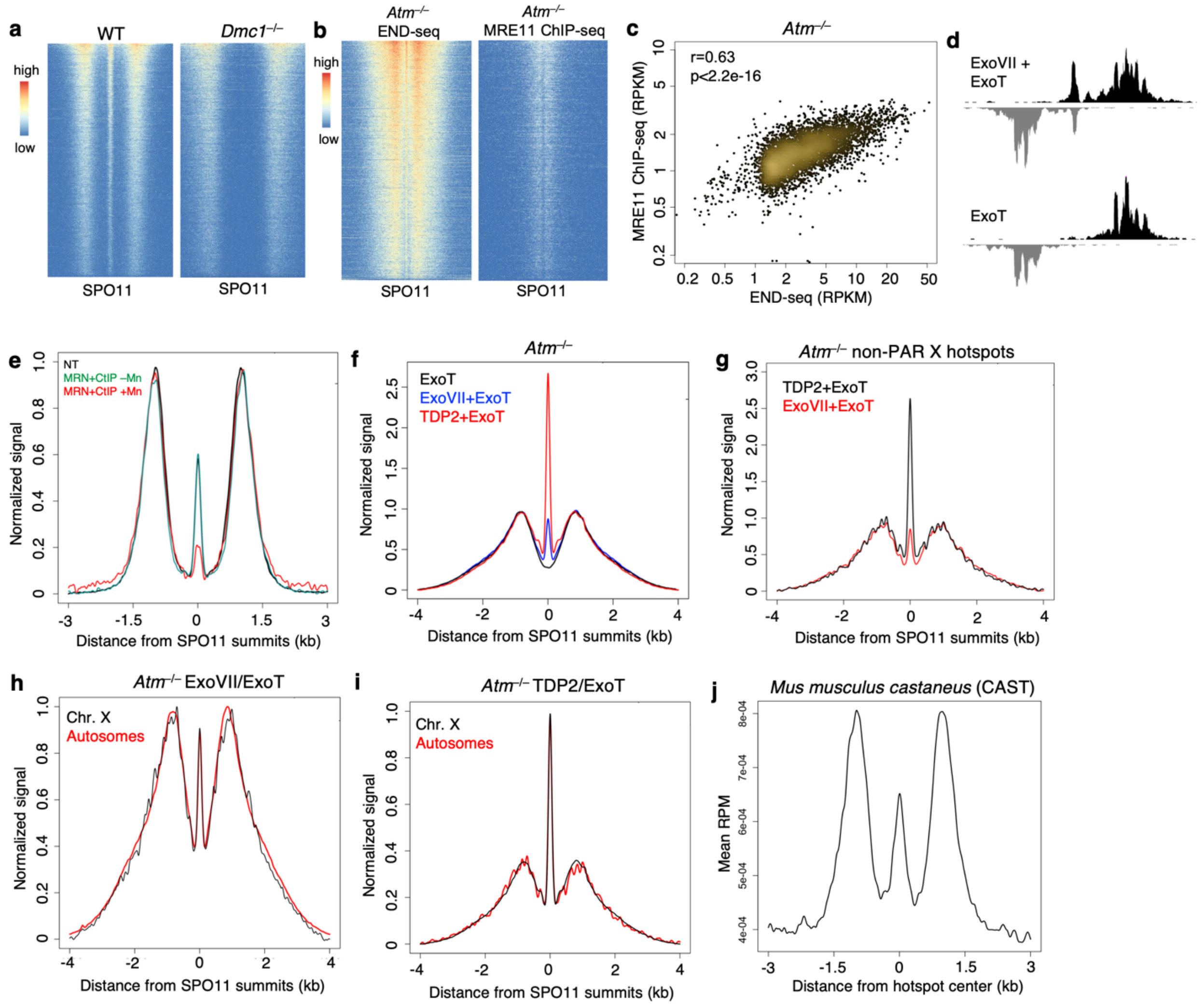

Supplementary Fig. 4 Detection of SPO11 cleavage complexes. (**a**) WT vs *Dmc1*$^{-/-}$ END-seq heatmap of top 5000 breaks showing lack of central signal and increased short- and long-range resection in DMC1-null background. All hotspots show absence of central signal by heatmap in a ±2kb window around SPO11 summits, ordered by total read count of WT END-seq. (**b**) Heatmaps of END-seq and MRE11 ChIP-seq in *Atm*$^{-/-}$ +/-5kb around SPO11 summits. Note that decreased MRE11 coverage is observed within NDR of *Atm*$^{-/-}$. (**c**) Correlation (Spearman's r) between END-seq intensity and MRE11 ChIP-seq per hotspot in *Atm*$^{-/-}$ spermatocytes. (**d**) A single hotspot example of END-seq with ExoVII/ExoT versus ExoT alone. SPO11 central peak detection entirely depends on ExoVII blunting. (**e**) Pretreatment with purified human MRN+CtIP reduces ExoVII+ExoT central peak detection (red) over no pretreatment (NT, black) and depends on the presence of manganese (green). One 12 dpp mouse used per condition. (**f**) Aggregate plot of *Atm*$^{-/-}$ END-seq signal (normalized to the same resection height) comparing ExoVII+ExoT, TDP2+ExoT, and ExoT alone. TDP2+ExoT is more efficient at detecting SPO11 cleavage complexes (SPO11cc) than ExoVII+ExoT processing. (**g**) Aggregate END-seq signal of *Atm*$^{-/-}$ non-PAR X chromosome hotspots (normalized to the same resection height) for TDP2+ExoT and ExoVII+ExoT. SPO11cc are present at non-PAR X chromosome hotspots in the absence of ATM. (**h-i**) Aggregate plot of *Atm*$^{-/-}$ ExoVII/ExoT (g) and TDP2/ExoT (h) END-seq signal (normalized to the same height) comparing SPO11cc intensity at non-PAR X

chromosome hotspots and hotspots on all autosomes. (**j**) Aggregate plot of END-seq signal of an adult CAST parent from the B6xCAST F1 hybrid crosses in Fig. 7d. Unlike the hybrid pups, CAST males have prominent central signal at hotspot centers, determined by DMC1 SSDS.

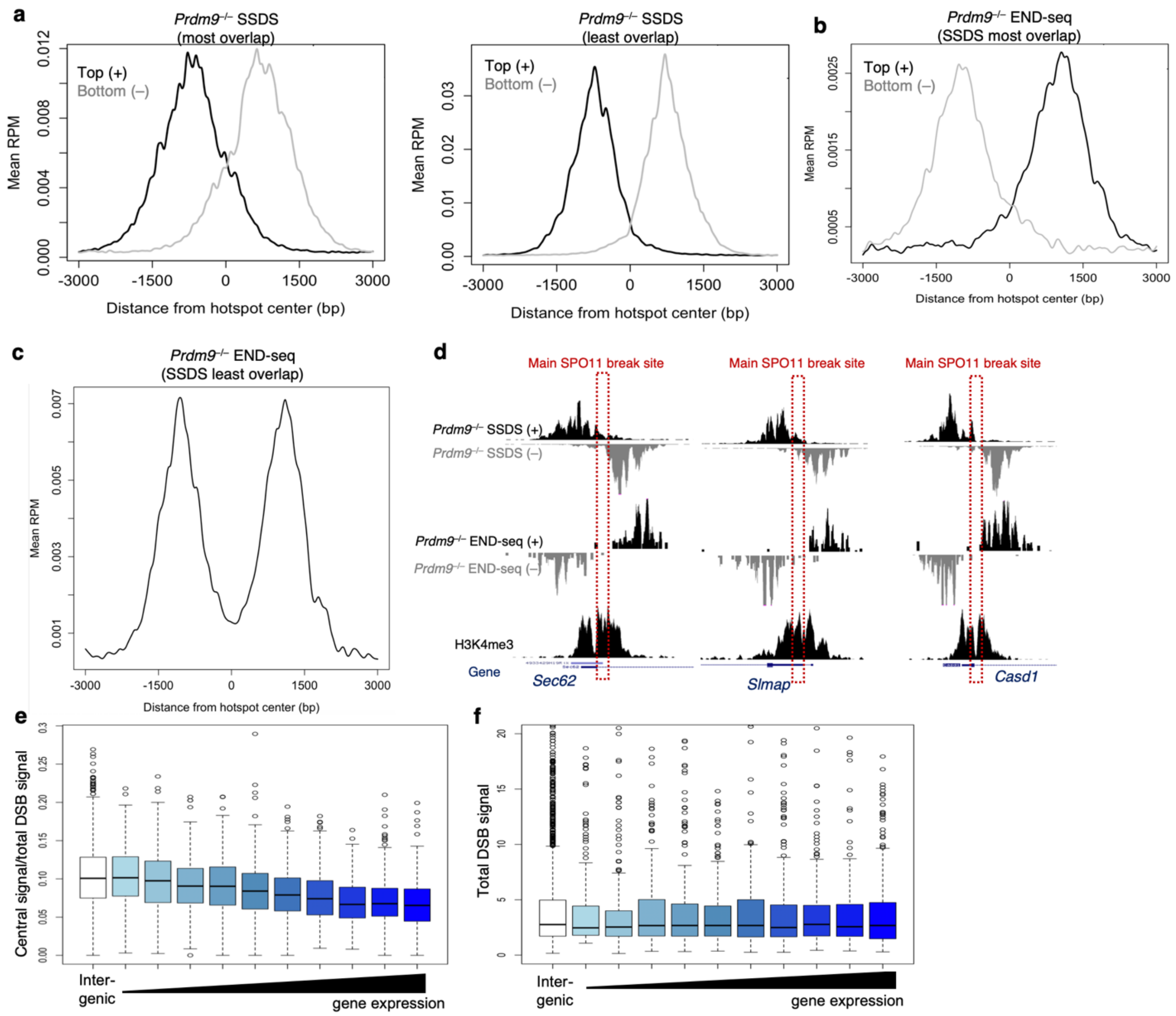

Supplementary Fig. 5 Characterization of PRDM9-independent hotspots. (**a**) Strand-separated aggregate plots of *Prdm9*$^{-/-}$ SSDS hotspots that have either the most top and bottom strand overlap (left, 375 hotspots) or least overlap (right, 199 hotspots), i.e. multiple SPO11 cut sites around the center (left) or a preferred SPO11 cut site (right). Overlap was determined by calculating, for each strand, the integration of signal from one side of the hotspot divided by the integration of total hotspot signal. "Most overlapped" hotspots have integration ratios of 0.75-0.85 (i.e. only 75-85% of total signal comes from one side of the hotspot) and "least overlapped" have ratios greater than 0.9. (**b**) Aggregate END-seq signal around *Prdm9*$^{-/-}$ SSDS hotspot centers at sites described as "most overlapped" in "a, left". At these sites in which there is no clear center for SPO11 cutting, END-seq resection signal from multiple, adjacent cut sites within the promoter results in ambiguous central signal detection. We therefore focused analyses on the "least overlapped" hotspots described in "a, right". (**c**) Aggregate plot of non-strand-separated *Prdm9*$^{-/-}$ END-seq around SSDS hotspot centers at the least strand overlapped SSDS sites shown in "a, right". (**d**) *Prdm9*$^{-/-}$ SSDS and END-seq tracks at additional default hotspots with minimal SSDS top and bottom strand overlap. (**e-f**) Boxplots comparing the ratio of WT END-seq central signal to total DSB END-seq signal per hotspot. Boxplots are categorized as either intergenic hotspots or genic hotspots (located within gene bodies) that are further categorized into ten

groups based on gene expression data. Central signal decreases proportionally with increasing gene expression (e), while total break intensity per hotspots is unaffected by transcription (f).
