# Supplementary material for: ATM and PRDM9 regulate SPO11-bound recombination intermediates during meiosis
Source: Nat Commun. 2020 Feb 12;11:857. doi: 10.1038/s41467-020-14654-w (PMC7016097; doi:10.1038/s41467-020-14654-w)
Supplement: Supplementary file 2 — Reporting Summary [file 41467_2020_14654_MOESM2_ESM.pdf]

nature research

Corresponding author(s): André Nussenzweig

Last updated by author(s): Jan 2, 2020

Reporting Summary

Nature Research wishes to improve the reproducibility of the work that we publish. This form provides structure for consistency and transparency in reporting. For further information on Nature Research policies, see Authors & Referees and the Editorial Policy Checklist.

Statistics

For all statistical analyses, confirm that the following items are present in the figure legend, table legend, main text, or Methods section.

n/a	Confirmed	
☐	☒	The exact sample size (*n*) for each experimental group/condition, given as a discrete number and unit of measurement
☐	☒	A statement on whether measurements were taken from distinct samples or whether the same sample was measured repeatedly
☐	☒	The statistical test(s) used AND whether they are one- or two-sided<br>*Only common tests should be described solely by name; describe more complex techniques in the Methods section.*
☒	☐	A description of all covariates tested
☐	☒	A description of any assumptions or corrections, such as tests of normality and adjustment for multiple comparisons
☐	☒	A full description of the statistical parameters including central tendency (e.g. means) or other basic estimates (e.g. regression coefficient) AND variation (e.g. standard deviation) or associated estimates of uncertainty (e.g. confidence intervals)
☐	☒	For null hypothesis testing, the test statistic (e.g. *F*, *t*, *r*) with confidence intervals, effect sizes, degrees of freedom and *P* value noted<br>*Give P values as exact values whenever suitable.*
☒	☐	For Bayesian analysis, information on the choice of priors and Markov chain Monte Carlo settings
☒	☐	For hierarchical and complex designs, identification of the appropriate level for tests and full reporting of outcomes
☐	☒	Estimates of effect sizes (e.g. Cohen's *d*, Pearson's *r*), indicating how they were calculated

Our web collection on statistics for biologists contains articles on many of the points above.

Software and code

Policy information about availability of computer code

Data collection	Data were collected using Illumina (NextSeq 550) next-generation sequencing platforms.
Data analysis	END-seq Data Analysis<br>Reads were aligned to the mouse (GRCm38p2/mm10) genome using Bowtie version 1.1.2 and 3 mismatches were allowed and the best strata for reads were kept with multiple alignments (-n 3 -k 1 -l 50). Functions ‘‘view’’ and ‘‘sort’’ of samtools (version 1.6) were used to convert and sort the mapping output to sorted bam file. Peaks were called using MACS 1.4.3. As the strand separated aggregate plots (Figure 2a) show ~2kb distance, END-seq peaks were called using the parameters: --shiftsize=1000, --nolambda, --nomodel and --keep-dup = all. Peaks with >2.5 fold-enrichment are kept and those within blacklisted regions (https://sites.google.com/site/anshulkundaje/projects/blacklists) were filtered.<br><br>Mouse Hybrid END-seq analysis<br>Single nucleotide polymorphisms (SNP) were annotated for CAST genome by the Sanger Mouse Genome Project based on B6 mouse reference genome (mm10). Only SNPs (n=20,668,274) passed all filter criteria were employed to generate an “N-masked” genome. 14,951 hotspots in B6xCAST hybrid genome were download from GSE75419.. Since Bowtie doesn’t allow gapped alignment for Ns, the hybrid END-seq reads were aligned by Bowtie2 version 2.3.5.1 with options -N 1 -k 1.<br><br>H3K4me3 and H3K36me3 ChIP-seq<br>For our H3K4me3 ChIP-seq and the public H3K36me3 ChIP-seq, reads were aligned to the mouse (GRCm38p2/mm10) genome using Bowtie version 1.1.2 with options -n 2 -m 1 -l 50.

For manuscripts utilizing custom algorithms or software that are central to the research but not yet described in published literature, software must be made available to editors/reviewers. We strongly encourage code deposition in a community repository (e.g. GitHub). See the Nature Research guidelines for submitting code & software for further information.

Data

Policy information about availability of data

All manuscripts must include a data availability statement. This statement should provide the following information, where applicable:

- Accession codes, unique identifiers, or web links for publicly available datasets
- A list of figures that have associated raw data
- A description of any restrictions on data availability

All raw and processed data are available in the GEO under accession number GSE138915.

Field-specific reporting

Please select the one below that is the best fit for your research. If you are not sure, read the appropriate sections before making your selection.

☒ Life sciences ☐ Behavioural & social sciences ☐ Ecological, evolutionary & environmental sciences

For a reference copy of the document with all sections, see nature.com/documents/nr-reporting-summary-flat.pdf

Life sciences study design

All studies must disclose on these points even when the disclosure is negative.

Sample size	Sample size (i.e. number of mice per library) was determined by FRiP score criteria set by ENCODE for ChIP-seq datasets. We found strong signal-to-noise using only 1 mouse per library, while 20 mice per library did not significantly change the data or conclusions. Thus, all libraries comprise 1-10 mice based on availability and breeding schemes.
Data exclusions	In analyses determining resection lengths, only top few hundred breaks were used, as slightly lower signal-to-noise at weaker breaks confounded our ability to accurate determine resection lengths.
Replication	We performed two replicates of END-seq libraries using 20 WT mice and found high reproducibility between them. Additionally, we found similarly strong reproducibility using only a single WT mouse. WT litter mate controls to KO studies also show reproducibility to pure B6 samples. For KO studies, we found strong reproducibility between two Atm-/- libraries, demonstrating that regardless of mouse number, background, and genotype, data are fully consistent and reproducible.
Randomization	Randomization was not necessary for our study as all mice were pooled for experiments to generate a single dataset.
Blinding	Blinding was not necessary for our study as all mice were pooled for experiments to generate a single dataset.

Reporting for specific materials, systems and methods

We require information from authors about some types of materials, experimental systems and methods used in many studies. Here, indicate whether each material, system or method listed is relevant to your study. If you are not sure if a list item applies to your research, read the appropriate section before selecting a response.

Materials & experimental systems

n/a	Involved in the study	
☐	☐	Antibodies
☐	☐	Eukaryotic cell lines
☐	☐	Palaeontology
☐	☐	Animals and other organisms
☐	☐	Human research participants
☐	☐	Clinical data

Methods

n/a	Involved in the study	
☐	☒	ChIP-seq
☐	☒	Flow cytometry
☐	☐	MRI-based neuroimaging

Antibodies

Antibodies used	*Describe all antibodies used in the study; as applicable, provide supplier name, catalog number, clone name, and lot number.*
Validation	*Describe the validation of each primary antibody for the species and application, noting any validation statements on the manufacturer's website, relevant citations, antibody profiles in online databases, or data provided in the manuscript.*

Eukaryotic cell lines

Policy information about cell lines

Cell line source(s)	*State the source of each cell line used.*
Authentication	*Describe the authentication procedures for each cell line used OR declare that none of the cell lines used were authenticated.*
Mycoplasma contamination	*Confirm that all cell lines tested negative for mycoplasma contamination OR describe the results of the testing for mycoplasma contamination OR declare that the cell lines were not tested for mycoplasma contamination.*
Commonly misidentified lines (See ICLAC register)	*Name any commonly misidentified cell lines used in the study and provide a rationale for their use.*

Palaeontology

Specimen provenance	*Provide provenance information for specimens and describe permits that were obtained for the work (including the name of the issuing authority, the date of issue, and any identifying information).*
Specimen deposition	*Indicate where the specimens have been deposited to permit free access by other researchers.*
Dating methods	*If new dates are provided, describe how they were obtained (e.g. collection, storage, sample pretreatment and measurement), where they were obtained (i.e. lab name), the calibration program and the protocol for quality assurance OR state that no new dates are provided.*

☐ Tick this box to confirm that the raw and calibrated dates are available in the paper or in Supplementary Information.

Animals and other organisms

Policy information about studies involving animals; ARRIVE guidelines recommended for reporting animal research

Laboratory animals	Males, 12-14 dpp, C57BL/6
Wild animals	Our study did not involve wild animals
Field-collected samples	Our study did not involve field-collected samples
Ethics oversight	All mouse breeding and experimentation followed protocols approved by the National Institutes of Health Institutional Animal Care and Use Committee

Note that full information on the approval of the study protocol must also be provided in the manuscript.

Human research participants

Policy information about studies involving human research participants

Population characteristics	*Describe the covariate-relevant population characteristics of the human research participants (e.g. age, gender, genotypic information, past and current diagnosis and treatment categories). If you filled out the behavioural & social sciences study design questions and have nothing to add here, write "See above."*
Recruitment	*Describe how participants were recruited. Outline any potential self-selection bias or other biases that may be present and how these are likely to impact results.*
Ethics oversight	*Identify the organization(s) that approved the study protocol.*

Note that full information on the approval of the study protocol must also be provided in the manuscript.

Clinical data

Policy information about clinical studies

All manuscripts should comply with the ICMJE guidelines for publication of clinical research and a completed CONSORT checklist must be included with all submissions.

Clinical trial registration	*Provide the trial registration number from ClinicalTrials.gov or an equivalent agency.*
Study protocol	*Note where the full trial protocol can be accessed OR if not available, explain why.*
Data collection	*Describe the settings and locales of data collection, noting the time periods of recruitment and data collection.*
Outcomes	*Describe how you pre-defined primary and secondary outcome measures and how you assessed these measures.*

ChIP-seq

Data deposition

☒ Confirm that both raw and final processed data have been deposited in a public database such as GEO.

☒ Confirm that you have deposited or provided access to graph files (e.g. BED files) for the called peaks.

Data access links<br>*May remain private before publication.*	https://www.ncbi.nlm.nih.gov/geo/query/acc.cgi?acc=GSE138915<br>Reviewer access token:oxstsymmxhobfup
Files in database submission	GSM4122801 WT H3K4me3 ChIP-seq<br>GSM4122802 ATM-/- H3K4me3 ChIP-seq<br>GSM4122803 WT MRE11 ChIP-seq<br>GSM4122804 ATM-/- MRE11 ChIP-seq
Genome browser session<br>(e.g. UCSC)	The bigwig files deposited in GEO database can be directly loaded into IGV or UCSC for visualization

Methodology

Replicates	No replicates.
Sequencing depth	single-end sequencing (75 bp) at 20 million reads per sample for H3K4me3 ChIP-seq and single-end sequencing (75 bp) at 50~60 million reads per sample for MRE11 ChIP-seq
Antibodies	Anti-MRE11, Novus Biologicals, NB100-142SS, polyclonal, lot R (unpurified rabbit sera)<br>Anti-H3K4me3, MilliporeSigma, 07-473, polyclonal, lot 3150888
Peak calling parameters	not relevant.
Data quality	fastqc was employed to examine the fastq quality.
Software	Bowtie version 1.1.2.

Flow Cytometry

Plots

Confirm that:

☒ The axis labels state the marker and fluorochrome used (e.g. CD4-FITC).

☒ The axis scales are clearly visible. Include numbers along axes only for bottom left plot of group (a 'group' is an analysis of identical markers).

☒ All plots are contour plots with outliers or pseudocolor plots.

☒ A numerical value for number of cells or percentage (with statistics) is provided.

Methodology

Sample preparation	Bulk single cell suspensions were isolated from whole testes and stained with Hoechst and propidium iodide for 3 hrs at 32C
Instrument	BD LSRFortessa
Software	FlowJo
Cell population abundance	cells were not sorted
Gating strategy	Cells were gated on FSC/SSC and FSC-H/FSC-W to gate on single cells

☐ Tick this box to confirm that a figure exemplifying the gating strategy is provided in the Supplementary Information.

Magnetic resonance imaging

Experimental design

Design type	*Indicate task or resting state; event-related or block design.*
Design specifications	*Specify the number of blocks, trials or experimental units per session and/or subject, and specify the length of each trial or block (if trials are blocked) and interval between trials.*

Behavioral performance measures | *State number and/or type of variables recorded (e.g. correct button press, response time) and what statistics were used to establish that the subjects were performing the task as expected (e.g. mean, range, and/or standard deviation across subjects).*

Acquisition

Imaging type(s) | *Specify: functional, structural, diffusion, perfusion.*

Field strength | *Specify in Tesla*

Sequence & imaging parameters | *Specify the pulse sequence type (gradient echo, spin echo, etc.), imaging type (EPI, spiral, etc.), field of view, matrix size, slice thickness, orientation and TE/TR/flip angle.*

Area of acquisition | *State whether a whole brain scan was used OR define the area of acquisition, describing how the region was determined.*

Diffusion MRI ☐ Used ☐ Not used

Preprocessing

Preprocessing software | *Provide detail on software version and revision number and on specific parameters (model/functions, brain extraction, segmentation, smoothing kernel size, etc.).*

Normalization | *If data were normalized/standardized, describe the approach(es): specify linear or non-linear and define image types used for transformation OR indicate that data were not normalized and explain rationale for lack of normalization.*

Normalization template | *Describe the template used for normalization/transformation, specifying subject space or group standardized space (e.g. original Talairach, MNI305, ICBM152) OR indicate that the data were not normalized.*

Noise and artifact removal | *Describe your procedure(s) for artifact and structured noise removal, specifying motion parameters, tissue signals and physiological signals (heart rate, respiration).*

Volume censoring | *Define your software and/or method and criteria for volume censoring, and state the extent of such censoring.*

Statistical modeling & inference

Model type and settings | *Specify type (mass univariate, multivariate, RSA, predictive, etc.) and describe essential details of the model at the first and second levels (e.g. fixed, random or mixed effects; drift or auto-correlation).*

Effect(s) tested | *Define precise effect in terms of the task or stimulus conditions instead of psychological concepts and indicate whether ANOVA or factorial designs were used.*

Specify type of analysis: ☐ Whole brain ☐ ROI-based ☐ Both

Statistic type for inference
(See Eklund et al. 2016) | *Specify voxel-wise or cluster-wise and report all relevant parameters for cluster-wise methods.*

Correction | *Describe the type of correction and how it is obtained for multiple comparisons (e.g. FWE, FDR, permutation or Monte Carlo).*

Models & analysis

n/a	Involved in the study
☐	☐ Functional and/or effective connectivity
☐	☐ Graph analysis
☐	☐ Multivariate modeling or predictive analysis

Functional and/or effective connectivity | *Report the measures of dependence used and the model details (e.g. Pearson correlation, partial correlation, mutual information).*

Graph analysis | *Report the dependent variable and connectivity measure, specifying weighted graph or binarized graph, subject- or group-level, and the global and/or node summaries used (e.g. clustering coefficient, efficiency, etc.).*

Multivariate modeling and predictive analysis | *Specify independent variables, features extraction and dimension reduction, model, training and evaluation metrics.*
