# Supplementary material for: ATM and PRDM9 regulate SPO11-bound recombination intermediates during meiosis
Source: Nat Commun. 2020 Feb 12;11:857. doi: 10.1038/s41467-020-14654-w (PMC7016097; doi:10.1038/s41467-020-14654-w)
Supplement: Supplementary file 3 — Description of Additional Supplementary Files [file 41467_2020_14654_MOESM3_ESM.pdf]

Description of Additional Supplementary Files

File Name: Supplementary Data 1
Description: Peaks called from WT mice END-seq.

File Name: Supplementary Data 2
Description: Peaks called from Atm-/- mice END-seq.
